# ﻿Phylogenetic diversity of *Colletotrichum* species (Sordariomycetes, Glomerellales, Glomerellaceae) associated with plant diseases in Thailand

**DOI:** 10.3897/mycokeys.119.152323

**Published:** 2025-07-03

**Authors:** Deecksha Gomdola, Rajesh Jeewon, Eric H.C. McKenzie, Ruvishika S. Jayawardena, Fatimah Al-Otibi, Xia Tang, Yong Wang, Kevin D. Hyde, Li Fu

**Affiliations:** 1 Shandong Institute of Pomology, Tai'an, Shandong 271000, China; 2 School of Science, Mae Fah Luang University, Chiang Rai 57100, Thailand; 3 Center of Excellence in Fungal Research, Mae Fah Luang University, Chiang Rai 57100, Thailand; 4 Department of Plant Pathology, College of Agriculture, Guizhou University, Guiyang, Guizhou 550025, China; 5 Department of Health Sciences, Faculty of Medicine and Health Sciences, University of Mauritius, Réduit 80837, Mauritius; 6 Department of Zoology, College of Science, King Saud University, Riyadh, Saudi Arabia; 7 Manaaki Whenua-Landcare Research, Private Mail Bag, 92170, Auckland, New Zealand; 8 Department of Botany and Microbiology, College of Science, King Saud University, P.O. Box 22452, Riyadh 11495, Saudi Arabia

**Keywords:** 2 novel species, 13 new host records, asexual, plant pathogens, sexual, species complexes, taxonomy

## Abstract

*Colletotrichum* is a cosmopolitan fungal genus, and its species are known for their important role as plant pathogens. They also occur as endophytes and saprobes. The genus comprises species complexes, many of which include cryptic species that are morphologically similar but phylogenetically distinct. *Colletotrichum* is characterized by globose to subglobose ascomata and acervular conidiomata and hyaline, aseptate ascospores and conidia, although a few species produce septate ascospores. Ascospores are typically cylindrical, oval, or fusiform, while conidia are mostly ellipsoidal to ovoid. Many *Colletotrichum* species also produce appressoria, which are formed at the tips of germ tubes or directly from the mycelium. In this study, we collected diseased leaves and pods with spots, blight, and blotches from Chiang Mai, Chiang Rai, and Tak provinces in Thailand. For species identification and delimitation, we employed a biphasic approach incorporating morphological data and multigene phylogenetic analyses of the locus of the internal transcribed spacer (ITS, nuclear rDNA consisting of ITS1-5.8S-ITS2) and the genes encoding for glyceraldehyde-3-phosphate dehydrogenase (*GAPDH*), chitin synthase 1 (*CHS*1), actin (*ACT*), beta-tubulin 2 (*TUB*2), histone (*H*3), and calmodulin (*CAM*). We obtained 20 *Colletotrichum* isolates representing eight species, among which we propose two new species: *Colletotrichumdendrobii* (from *Dendrobium* sp.) and *C.musichiangmaiense* (from *Musa* sp.). We also establish 13 new host records (for six species): *C.castaneae* (on *Jasminumgrandiflorum*), *C.chrysophilum* (on *Terminalia* sp.), *C.endophyticum* (on *Artocarpusheterophyllus*, *Begonialuxurians*, *Cassia* sp., *Castanopsis* sp., *Ficusauriculata*, and *Schefflera* sp.), *C.fructicola* (on *Castanea* sp., *Hedychium* sp., and *Rhododendron* sp.), *C.jiangxiense* (on *Artocarpus* sp.), and *C.schimae* (on *Jasminum* sp.). In addition, this is the first study to present the sexual morph of *C.endophyticum*.

## ﻿Introduction

*Colletotrichum* was introduced by Corda (1831) and typified with *C.lineola*. It is the sole genus in Glomerellaceae (Glomerellales, Sordariomycetes, Ascomycetes, Fungi) (Jayawardena et al. 2021a; Hyde et al. 2024a). Its sexual morph was formerly classified under *Glomerella* (Marin-Felix et al. 2017), which is characterized by globose to subglobose, dark brown to black, ostiolate ascomata; unitunicate and cylindrical asci; and hyaline, aseptate ascospores that are smooth-walled, cylindrical to oval or fusiform with acute and/or rounded ends, although a few species, such as *C.gigasporum* and *C.taiwanense*, produce septate ascospores. Its asexual morph is characterized by acervular conidiomata, hyaline to pale brown conidiophores that are branched or unbranched, enteroblastic, hyaline to pale brown conidiogenous cells that are cylindrical to ellipsoidal or ampulliform, and hyaline, aseptate, smooth-walled, ellipsoidal to ovoid conidia that are straight or slightly flexuous, with some species producing curved, falcate, or lunate conidia, with a rounded or acute apex, and rounded or truncate base. Setae and appressoria may be present, with appressoria produced from germ tubes or directly from the mycelium (Damm et al. 2009; Jayawardena et al. 2016a, 2021a; Marin-Felix et al. 2017; Bhunjun et al. 2021; Liu et al. 2022).

*Colletotrichum* has a cosmopolitan distribution with a wide host range, colonizing a myriad of plants and displaying remarkable adaptability to different environments (Jayawardena et al. 2016a, 2020, 2021a, 2022; Armand et al. 2023; Talhinhas and Baroncelli 2023; Yang et al. 2023; Zhang et al. 2023a, 2023b; Aumentado et al. 2024; Hyde et al. 2024b; Sui et al. 2024). *Colletotrichum* species are predominantly pathogens, typically exhibiting a necrotrophic phase and, in some cases, a short biotrophic stage (O’Connell et al. 2012). Plant diseases caused by *Colletotrichum* taxa include anthracnose (sunken necrotic spots), spots, blights and blotches of leaves and fruits, as well as stem rot and dieback (Than et al. 2008a, b; Jayawardena et al. 2016a, 2021a; Marin-Felix et al. 2017). Some species can infect insects (Wynns et al. 2019; De Vivo et al. 2021) and humans (Hung et al. 2020; Paniz-Mondolfi et al. 2021). Besides being notorious pathogens, they are endophytes, epiphytes, and saprobes (Jayawardena et al. 2021a).

There are over 1000 epithets in Index Fungorum (2025), but only about 350 species are fully described with molecular data (Jayawardena et al. 2021a; Talhinhas and Baroncelli 2023). Based on the current classification system, they are distributed across 15 species complexes, with about 20 singleton species, that is, species not assigned to any complex (Bhunjun et al. 2021; Talhinhas and Baroncelli 2021, 2023; Sui et al. 2024). The species complexes are *C.acutatum*, *C.agaves*, *C.bambusicola*, *C.boninense*, *C.dematium*, *C.destructivum*, *C.dracaenophilum*, *C.gigasporum*, *C.gloeosporioides*, *C.graminicola-caudatum*, *C.magnum*, *C.orbiculare*, *C.orchidearum*, *C.spaethianum*, and *C.truncatum* (Jayawardena et al. 2021a; Talhinhas and Baroncelli 2023; Aumentado et al. 2024). The *C.caudatum* species complex is grouped within the *C.graminicola* species complex instead of forming a larger distinct clade (Marin-Felix et al. 2017; Jayawardena et al. 2020; Liu et al. 2022). Thus, Bhunjun et al. (2021) proposed combining the two complexes as the *C.graminicola-caudatum* species complex (Sui et al. 2024).

In this study, 20 *Colletotrichum* isolates representing eight species were obtained from diseased leaves and pods collected in Thailand. Detailed morphological examinations were conducted, and their taxonomic placement was confirmed using phylogenetic analyses based on concatenated gene trees incorporating the locus of the internal transcribed spacer (ITS, nuclear rDNA consisting of ITS1-5.8S-ITS2) and the genes encoding for glyceraldehyde-3-phosphate dehydrogenase (*GAPDH*), chitin synthase 1 (*CHS*1), actin (*ACT*), beta-tubulin 2 (*TUB*2), histone (*H*3), and calmodulin (*CAM*). Two new species are proposed: *Colletotrichumdendrobii*, associated with pod blight of *Dendrobium* sp. and placed in the *C.spaethianum* species complex, and *C.musichiangmaiense*, a singleton species associated with leaf blight of *Musa* sp. Additionally, 13 new host records are established for six species: *C.castaneae*, *C.chrysophilum*, *C.endophyticum*, *C.fructicola*, *C.jiangxiense*, and *C.schimae*. The sexual morph of *C.endophyticum* is herein reported for the first time. Furthermore, an updated phylogenetic tree is provided, incorporating all *Colletotrichum* taxa with DNA sequence data across species complexes, alongside a revised count of species distributed within each complex and the number of singleton species.

This research enhances the understanding of plant pathogens that impact crop yield and food security through the development of accurate fungal diagnostics to support sustainable agriculture and by improving knowledge of plant-fungal interactions, biodiversity, and ecosystem health.

## ﻿Materials and methods

### ﻿Sample collection, examination, and material deposition

Diseased leaves and pods displaying symptoms such as spots, blight, and blotches were collected from Chiang Mai, Chiang Rai, and Tak Provinces in Thailand. Specimens were stored in plastic bags and brought to the laboratory for further examination. Micro- and macro-morphological characteristics were examined with a Motic SMZ 168 Series stereomicroscope. Single spore and tissue isolation techniques following the guidelines outlined in Senanayake et al. (2020) were used to isolate the fungi observed. Single spores and individual hyphal tips were transferred aseptically to fresh potato dextrose agar (PDA; 39 g/L) and malt extract agar plates (MEA; 50 g/L). Pure cultures were incubated at 25 °C, and sporulated cultures were observed after 28 d. Images were captured using a Canon 750D camera (Canon, Tokyo, Japan) affixed to a Nikon ECLIPSE E600 compound microscope (Nikon, Tokyo, Japan). Photo plates were constructed with Adobe Photoshop CS6 version 2020 (Adobe Systems, USA), and measurements for each feature were obtained using Tarosoft® Image Frame Work (version 0.97).

Dried cultures are stored in the Mae Fah Luang University Herbarium (MFLU), and living cultures are deposited in the Mae Fah Luang University Culture Collection (MFLUCC), Thailand. FacesofFungi numbers (FoF) for isolates that previously lacked one are provided here (Jayasiri et al. 2015), and Index Fungorum (IF) numbers are assigned to the novel species (Index Fungorum, 2025). The descriptions, illustrations, and phylogenetic trees have been updated in the GMS microfungi database (https://gmsmicrofungi.org/) (Chaiwan et al. 2021).

### ﻿DNA extraction, PCR amplification, and sequencing

Genomic DNA was extracted from axenic cultures using the BIOMIGA Fungus Genomic DNA Extraction Kit (San Diego, CA, USA), following the manufacturer’s protocols. Seven gene regions, the locus of the internal transcribed spacer (ITS, nuclear rDNA consisting of ITS1-5.8S-ITS2), and the genes encoding for glyceraldehyde-3-phosphate dehydrogenase (*GAPDH*), chitin synthase 1 (*CHS*1), actin (*ACT*), beta-tubulin 2 (*TUB*2), histone (*H*3), and calmodulin (*CAM*) were amplified and sequenced to identify the *Colletotrichum* isolates at the species level. The following primer pairs were used: ITS1/ITS4 for ITS (White et al. 1990), GDF1/GDR1 for *GAPDH* (Guerber et al. 2003), CHS-79F/CHS-345R for *CHS*1 (Carbone and Kohn 1999), ACT-512F/ACT-783R for *act* (Carbone and Kohn 1999), T1/Bt2b for *TUB*2 (Glass and Donaldson 1995), CYLH3F/CYLH3R for *H3* (Liu et al. 2022), and CL1C/CL2C for *CAM* (Weir et al. 2012).

The polymerase chain reaction (PCR) mixture was prepared to a final volume of 20 µL, which included 10 µL of PCR master mix, 1 µL each of forward and reverse primers (10 µM stock concentration), 7 µL of double-distilled water, and 1 µL of template DNA. The PCR program consisted of an initial denaturation at 95 °C for 3 min, followed by 40 cycles of denaturation at 95 °C for 45 s, annealing at 55 °C for 50 s for ITS, 52 °C for 1 min for *GAPDH* and *ACT*, and 58 °C for 1 min and 30 s for *CHS*1, *TUB*2, *CAM*, and *H*3, with an extension step at 72 °C for 2 min and a final extension at 72 °C for 10 min. Purification and sequencing of the PCR amplicons for the forward and reverse directions were performed by Sangon Biotech (Shanghai) Co., China.

### ﻿Phylogenetic analysis

The quality of sequences was verified using DNA Baser Assembler, and unreadable bases at each end were trimmed manually. Sequences were subjected to BLAST searches in NCBI, and sequence data of other species were retrieved from GenBank (Suppl. material 1). Consensus sequences were obtained using SeqMan (DNAStar, Madison, Wisconsin, USA). Individual gene datasets were aligned using MAFFT version 7 with default settings (https://mafft.cbrc.jp/alignment/server/) (Katoh et al. 2019) and trimmed using trimAl (Capella-Gutiérrez et al. 2009). The trimmed datasets were combined using SequenceMatrix (Vaidya et al. 2011). Four phylogenetic trees were generated based on four different datasets. Dataset 1 included a broader taxon sampling, encompassing all *Colletotrichum* species across all species complexes. The remaining datasets focused on specific species complexes, given that we recovered isolates that belong to those complexes. Dataset 2 included all species from the *C.gloeosporioides* species complex, dataset 3 included all species from the *C.spaethianum* species complex, and dataset 4 included all species from the *C.acutatum* species complex.

Maximum likelihood (ML) phylogenetic analysis was performed in the IQ-TREE web server (https://iqtree.cibiv.univie.ac.at) by applying the default parameters and 1000 ultrafast bootstrap replicates (Nguyen et al. 2015). Bayesian inference (BI) was conducted using MrBayes on XSEDE (version 3.2.7a) in the online CIPRES Science Gateway version 3.3 (https://www.phylo.org/portal2) (Huelsenbeck et al. 2001; Ronquist and Huelsenbeck 2003; Miller et al. 2010). Nucleotide substitution models generated automatically in the ML log file were selected as the best-fit models based on the Bayesian information criterion (BIC) and are as follows: GTR+F+I+G4 for ITS, TIM3e+I+G4 for *GAPDH*, TIMe+I+G4 for *CHS*1, HKY+F+I+G4 for *ACT*, TNe+I+G4 for *TUB*2, TN+F+I+G4 for *H*3, *and* TIM+F+I+G4 for *CAM*. Markov chain Monte Carlo (MCMC) sampling was applied to obtain posterior probabilities (PP). Four Markov chains with trees being sampled every 1000 generations were simultaneously run for 50,000,000, 80,000,000, 25,000,000, and 55,000,000 generations for trees 1 to 4, respectively. A burn-in of 20% was implemented, with the remaining 80% used to calculate the PP of the consensus trees.

The alignments are deposited in Figshare (https://doi.org/10.6084/m9.figshare.28377707). Phylogenetic trees were visualized in FigTree version 1.4.4 (Rambaut and Drummond 2014) and edited using Microsoft PowerPoint (version 2016), Inkspace version 1.2.2 (Harrington 2005), and the tvBOT web application (Xie et al. 2023).

## ﻿Results

### ﻿Phylogenetic analyses

**Figure 1.**
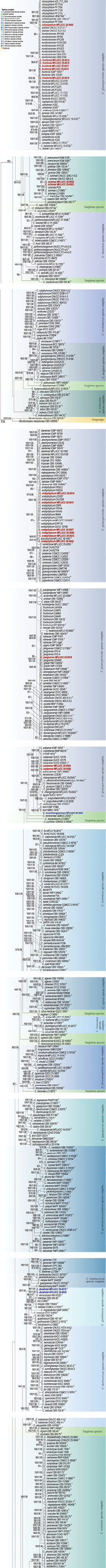
Maximum likelihood analysis (IQ-tree) based on a combined dataset of ITS, *GAPDH*, *CHS*1, *ACT*, *TUB*2, *H*3, and *CAM* sequences from dataset 1, which includes all *Colletotrichum* species. Bootstrap support values (ML ≥ 80%) and Bayesian posterior probabilities (PP ≥ 0.95) are given above the branches or near the nodes as ML/PP. Hyphens (--) indicate bootstrap support values below 80% for ML and posterior probabilities below 0.95. The tree is rooted with *Monilochaetesinfuscans* (CBS 869.96) and *M.melastomae* (CBS145059). Type, ex-type, and reference species are denoted with ^T^. Our isolates are in bold red font, while our new species are in bold blue font. Different color blocks represent distinct species complexes, with a single color used to denote singleton species.

**Figure 2.**
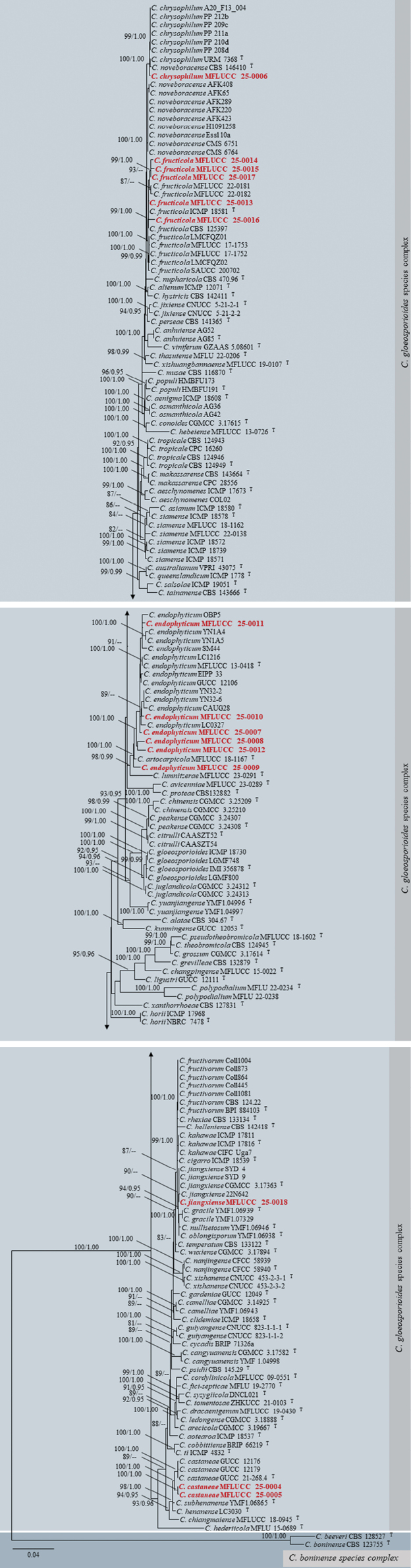
Maximum likelihood analysis (IQ-tree) based on a combined dataset of ITS, *GAPDH*, *CHS*1, *ACT*, *TUB*2, *H*3, and *CAM* sequences from dataset 2, which includes all species in the *C.gloeosporioides* species complex. Bootstrap support values (ML ≥ 80%) and Bayesian posterior probabilities (PP ≥ 0.95) are given above the branches or near the nodes as ML/PP. Hyphens (--) indicate bootstrap support values below 80% for ML and posterior probabilities below 0.95. The tree is rooted with *C.beeveri* (CBS 128527) and *C.boninense* (CBS 123755), belonging to the *C.boninense* species complex. Type, ex-type, and reference species are denoted with ^T^. Our isolates are in bold red font. Different color blocks represent distinct species complexes.

**Figure 3.**
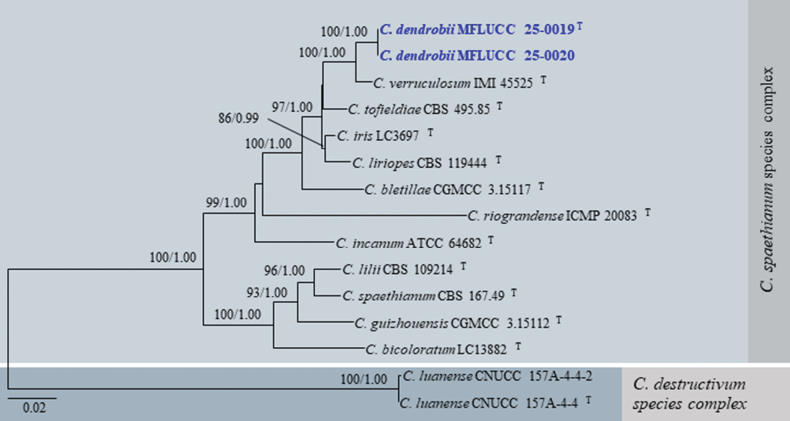
Maximum likelihood analysis (IQ-tree) based on a combined dataset of ITS, *GAPDH*, *CHS*1, *ACT*, *TUB*2, *H*3, and *CAM* sequences from dataset 3, which includes all species in the *C.spaethianum* species complex. Bootstrap support values (ML ≥ 80%) and Bayesian posterior probabilities (PP ≥ 0.95) are given above the branches or near the nodes as ML/PP. Hyphens (--) indicate bootstrap support values below 80% for ML and posterior probabilities below 0.95. The tree is rooted with *C.luanense* (CNUCC 157A-4-4 and CNUCC 157A-4-4-2), belonging to the *C.destructivum* species complex. Type, ex-type, and reference species are denoted with ^T^. Our new species are in bold blue font. Different color blocks represent distinct species complexes.

**Figure 4.**
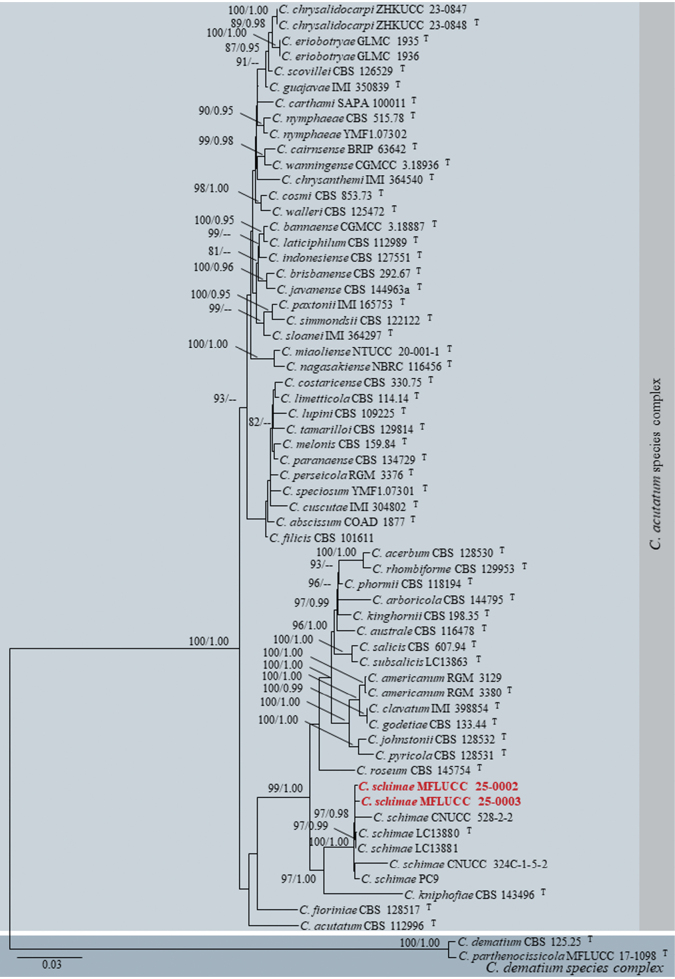
Maximum likelihood analysis (IQ-tree) based on a combined dataset of ITS, *GAPDH*, *CHS*1, *ACT*, *TUB*2, *H*3, and *CAM* sequences from dataset 4, which includes all species in the *Colletotrichumacutatum* species complex. Bootstrap support values (ML ≥ 80%) and Bayesian posterior probabilities (PP ≥ 0.95) are given above the branches or near the nodes as ML/PP. Hyphens (--) indicate bootstrap support values below 80% for ML and posterior probabilities below 0.95. The tree is rooted with *C.dematium* (CBS 125.25) and *C.parthenocissicola* (MFLUCC 17-1098), belonging to the *C.dematium* species complex. Type, ex-type, and reference species are denoted with ^T^. Our isolates are in bold red font. Different color blocks represent distinct species complexes.

Dataset 1 consisted of 2920 characters (ITS = 1–524, *GAPDH* = 525–777, *CHS*1 = 778–1059, *ACT* = 1060–1315, *TUB*2 = 1316–1813, *H*3 = 1814–2189, and *CAM* = 2190–2920). Dataset 2 consisted of 3137 characters (ITS = 1–534, *GAPDH* = 535–797, *CHS*1 = 798–1087, *ACT* = 1088–1354, *TUB*2 = 1355–2022, *H*3 = 2023–2406, and *CAM* = 2407–3137). Dataset 3 consisted of 2920 characters (ITS = 1–519, *GAPDH* = 520–773, *CHS*1 = 774–1024, *ACT* = 1025–1261, *TUB*2 = 1262–1751, *H*3 = 1752–2122, and *CAM* = 2123–2920). Dataset 4 consisted of 2981 characters (ITS = 1–538, *GAPDH* = 539–788, *CHS*1 = 789–1070, *ACT* = 1071–1316, *TUB*2 = 1317–1806, *H*3 = 1807–2190, and *CAM* = 2191–2981). The log-likelihood of the consensus trees was -83865.668, -18946.853, -7997.184, and -11551.837 for trees 1–4, respectively (Figs 1–4).

### ﻿Taxonomy

#### ﻿*Colletotrichumacutatum* species complex

##### ﻿ Colletotrichum schimae

Taxon classificationFungiGlomerellalesGlomerellaceae

F. Liu, W.P. Wu & L. Cai, in Liu et al. Stud. Mycol. 101:38 (2022)

6D5C937C-345A-5A7B-9CBA-B4C87DB7B043

Index Fungorum: IF841391

Facesoffungi Number: FoF17279

Figs 4 , 5

###### Description.

Associated with leaf spots of *Jasminum* sp. ***Leaf spots*** circular, pale brown to brown. Sexual morph: Not observed. Asexual morph on PDA: ***Conidiomata*** 200–1000 µm diam. (x̄ = 400 µm, n = 10), semi-immersed, scattered or segregated, globose to subglobose, dark brown to black, exuding glistening yellowish to orange conidial mass. ***Setae*** not observed. ***Conidiophores*** formed directly from mycelium, hyaline, sometimes septate. ***Conidiogenous cells*** 16–45 × 2–4.5 µm (x̄ = 32 × 3.5 µm, n = 10), formed terminally or laterally on hyphae, hyaline, cylindrical, solitary or branched, straight or flexuous, tapering towards the apex. ***Conidia*** 9–15 × 2.5–4 µm (x̄ = 11.5 × 3.5 µm, n = 30; L/W ratio = 3.3), hyaline, cylindrical to fusiform, smooth-walled, guttulate, aseptate, mostly with acute ends. ***Appressoria*** not observed.

###### Culture characteristics.

Colonies on PDA reaching approximately 40 mm diam. after 7 d of incubation at 25 °C; mycelium initially white, becoming yellowish to olivaceous brown with age; elevation flat, with an entire margin.

###### Specimens examined.

Thailand • Chiang Mai Province, Doi Inthanon National Park, Kew Mae Pan nature trail, associated with leaf spots of *Jasminum* sp. (Oleaceae), 20 Oct 2021, D. Gomdola DGJas1(L2)-A (MFLU 25-0001, BHH 50476), living culture MFLUCC 25-0002; DGJas1(L3)-B (MFLU 25-0002, BHH 50478), living culture MFLUCC 25-0003.

**Figure 5.**
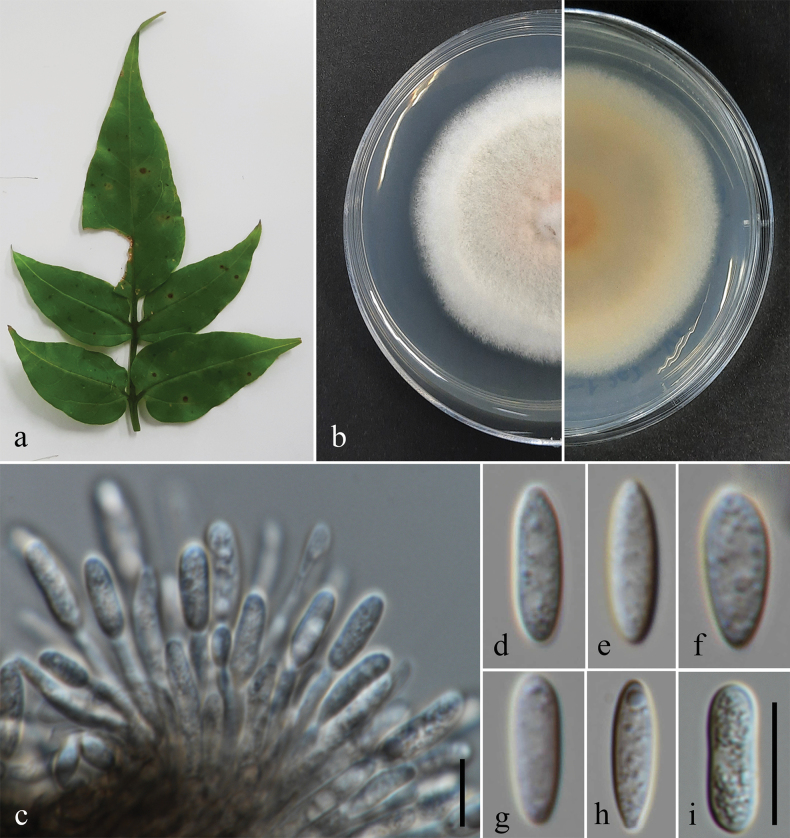
*Colletotrichumschimae*. **a.** Leaf spots (*Jasminum* sp.); **b.** Top and reverse of colony on PDA after 7 d; **c.** Conidiogenesis and developing conidia; **d–i.** Conidia. Scale bars: 10 μm. The scale bar (**i**) applies to **d–h**.

###### GenBank accession numbers.

MFLUCC 25-0002; ITS = PV263289; *GAPDH* = PV290895; *CHS*1 = PV274246; *ACT* = PV297872; and *TUB*2 = PV295615; MFLUCC 25-0003; ITS = PV263290; *GAPDH* = PV290896; *CHS*1 = PV274247; *ACT* = PV297873; and *TUB*2 = PV295616.

###### Known hosts, distributions, and lifestyles.

***Endophytic*** on *Schima* sp. in China (Liu et al. 2022).

***Pathogenic*** on leaves of Camelliasinensisvar.assamica and *Ilexchinensis* in China (Sui et al. 2024).

Associated with leaf spots of *Jasminum* sp. in Thailand (this study).

###### Notes.

Our isolates (MFLUCC 25-0002 and MFLUCC 25-0003) grouped with *Colletotrichumschimae* (LC13880, LC13881, PC9, CNUCC 324C-1-5-2, and CNUCC 528-2-2) with 97% ML and 0.98 PP support (Fig. 4). In our phylogenetic analyses, *C.schimae* is positioned within the *C.acutatum* species complex (Figs 1, 4), consistent with the findings of Liu et al. (2022) and Sui et al. (2024). Nucleotide sequence comparisons between our two isolates and *C.schimae* (LC13880) showed no differences in ITS (494 base pairs, bp), *GAPDH* (215 bp), and *TUB*2 (490 bp) regions. However, a 0.8% sequence divergence (2/245 bp) was observed in the *CHS*1 gene between our isolates and the ex-type of *C.schimae* (LC13880). For *ACT*, no differences were found between MFLUCC 25-0002 and *C.schimae* (LC13880), but a 1.9% divergence (4/208 bp) was observed between MFLUCC 25-0003 and *C.schimae* (LC13880).

MFLUCC 25-0002 and MFLUCC 25-0003 morphologically resemble the ex-type of *C.schimae* (LC13880) with hyaline, cylindrical conidiogenous cells and hyaline, smooth-walled, guttulate, and cylindrical to fusoid conidia with acute ends (Liu et al. 2022). Additionally, the conidial length-to-width ratio (L/W) of our isolates is similar to that of *C.schimae* (LC13880) (L/W ratio = 3.3 vs. 3.3).

Based on phylogenetic and morphological species concepts, we identify our isolates as *Colletotrichumschimae*. This study represents the first report of *C.schimae* associated with leaf spots on *Jasminum* sp. and establishes a new geographical record in Thailand.

#### ﻿*Colletotrichumgloeosporioides* species complex

##### ﻿ Colletotrichum castaneae

Taxon classificationFungiGlomerellalesGlomerellaceae

Y.Q. Yang, Q. Zhang, K.D. Hyde & Yong Wang bis, in Zhang et al., Mycosphere 14(2):24 (2023)

7C62D879-C8CF-52A9-80D3-234D95894913

Index Fungorum: IF559998

Facesoffungi Number: FoF05681

Figs 2 , 6

###### Description.

Associated with leaf spots of *Jasminumgrandiflorum*. ***Leaf spots*** irregular, pale brown to brown. Sexual morph: Not observed. Asexual morph on PDA: ***Conidiomata*** 150–500 µm diam. (x̄ = 250 µm, n = 5), semi-immersed, scattered or segregated, globose to subglobose, dark brown to black, exuding glistening yellowish to orange conidial mass. ***Setae*** not observed. ***Conidiophores*** hyaline. ***Conidiogenous cells*** 6.5–28 × 2–5 µm (x̄ = 18.6 × 3.1 µm, n = 10), hyaline, cylindrical to ampulliform, solitary or branched, straight or flexuous, tapering towards the apex. ***Conidia*** 13.5–19.5 × 4–6 µm (x̄ = 16.1 × 5 µm, n = 30; L/W ratio = 3.2), hyaline, cylindrical, straight, smooth-walled, guttulate, aseptate, mostly with rounded ends. ***Appressoria*** not observed.

###### Culture characteristics.

Colonies on PDA reaching approximately 65 mm diam. after 7 d of incubation at 25 °C; mycelium initially white, becoming yellowish to olivaceous brown with age; elevation flat, aerial, and filamentous with an entire or undulate margin.

**Figure 6.**
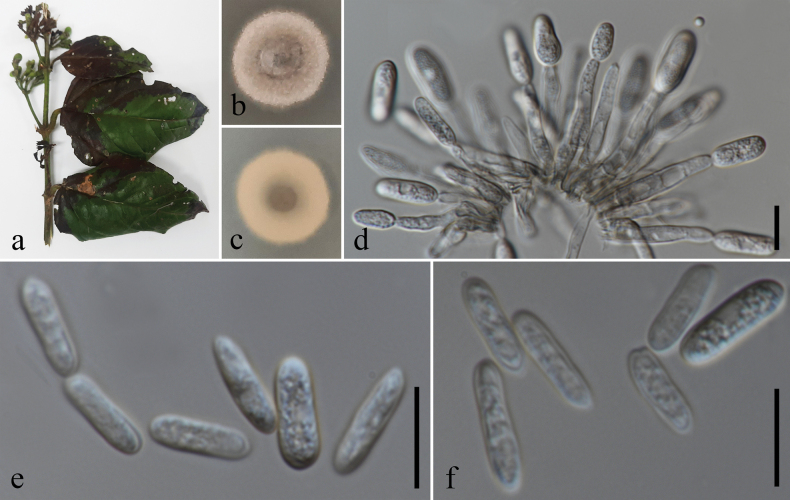
*Colletotrichumcastaneae*. **a.** Leaf spots (*Jasminumgrandiflorum*); **b, c.** Top and reverse of colony on PDA after 3 d **d** Conidiogenesis and developing conidia; **e, f.** Conidia. Scale bars: 10 μm.

###### Specimens examined.

Thailand • Chiang Mai Province, Doi Inthanon National Park, Kew Mae Pan nature trail, associated with leaf spots of *Jasminumgrandiflorum* (Oleaceae), 20 Oct 2021, D. Gomdola DGJas3(L6)-C (MFLU 25-0003, BHH 50477), living culture MFLUCC 25-0004; DGJas3(L9)-B (MFLU 25-0004, BHH 50479), living culture MFLUCC 25-0005.

###### GenBank accession numbers.

MFLUCC 25-0004; ITS = PV263291; *GAPDH* = PV290897; *CHS*1 = PV274248; *ACT* = PV297874; and *TUB*2 = PV295617; MFLUCC 25-0005; ITS = PV263292; *GAPDH* = PV290898; *CHS*1 = PV274249; *ACT* = PV297875; and *TUB*2 = PV295618.

###### Known hosts, distributions, and lifestyles.

Associated with leaf spots of *Castaneamollissima* in China (Zhang et al. 2023a) and *Jasminumgrandiflorum* in Thailand (this study).

###### Notes.

Based on the phylogenetic analyses, our isolates (MFLUCC 25-0004 and MFLUCC 25-0005) grouped with 100% ML and 1.00 PP support. This sub-clade grouped with *Colletotrichumcastaneae* (GUCC 21268.4, GUCC 12179, and GUCC 12176) with 100% ML and 1.00 PP support (Fig. 2). As per our findings, *C.castaneae* is located within the *C.gloeosporioides* species complex (Figs 1, 2), consistent with the study of Zhang et al. (2023a). No nucleotide differences were observed in the ITS region (526 bp) between our isolates and *C.castaneae* (GUCC 21268.4). The following sequence divergences were noted across other regions: 0.4% in *GAPDH* (1/228 bp), 0.9% in *ACT* (2/231 bp), 1.3% in *CHS*1 (3/225 bp), and 0.6% in *TUB*2 (3/495 bp).

MFLUCC 25-0004 and MFLUCC 25-0005 morphologically resemble the ex-type of *C.castaneae* (GUCC 21268.4) with hyaline, ampulliform to obclavate conidiogenous cells and hyaline, aseptate, smooth-walled, and cylindrical conidia, mostly with rounded ends (Zhang et al. 2023a). The conidial L/W ratio of our isolates is also similar to that of *C.castaneae* (GUCC 21268.4) (L/W ratio = 3.4 vs. 3.2).

Based on phylogenetic and morphological species concepts, we identify our isolates as *Colletotrichumcastaneae*. This study represents the first report of *C.castaneae* associated with leaf spots on *Jasminumgrandiflorum* and establishes a new geographical record in Thailand.

##### ﻿ Colletotrichum chrysophilum

Taxon classificationFungiGlomerellalesGlomerellaceae

W.A.S. Vieira, W.G. Lima, M.P.S. Câmara & V.P. Doyle, Mycologia 109(6): 927 (2017)

745EE575-890A-56F2-AA01-321A75D10542

Index Fungorum: IF821899

Facesoffungi Number: FoF17278

Figs 2 , 7

###### Description.

Associated with leaf spots of *Terminalia* sp. ***Leaf spots*** irregular or oval, pale brown, surrounded with a dark brown margin. Sexual morph: Not observed. Asexual morph on substrate: ***Conidiomata*** 70–120 × 60–120 µm (x̄ = 94.5 × 96 µm, n = 10), semi-immersed, scattered, globose to subglobose, black, sometimes erumpent. ***Setae*** not observed. ***Conidiomatal wall*** 8.5–21 µm thick (x̄ = 14.6 µm, n = 10), consisting of 3–5 layers of thick-walled pseudoparenchymatous cells of ***textura angularis***, outer layers dark brown, inner layer pale brown to hyaline. Asexual morph on PDA: ***Conidiophores*** hyaline, smooth-walled, aseptate, unbranched. ***Conidiogenous cells*** 16–22 × 3–4.5 µm (x̄ = 18.9 × 3.6 µm, n = 10), hyaline, cylindrical to ampulliform, straight or flexuous, tapering towards the apex. ***Conidia*** 13–19 × 4.5–6 µm (x̄ = 15.8 × 5.1 µm, n = 30; L/W ratio = 3.1), hyaline, cylindrical or oblong, smooth-walled, guttulate, aseptate, mostly with rounded ends. ***Appressoria*** 7–12 × 7–13 µm (x̄ = 10.9 × 10.5 µm, n = 5), hyaline, single-celled, globose to subglobose or irregular, smooth-walled.

**Figure 7.**
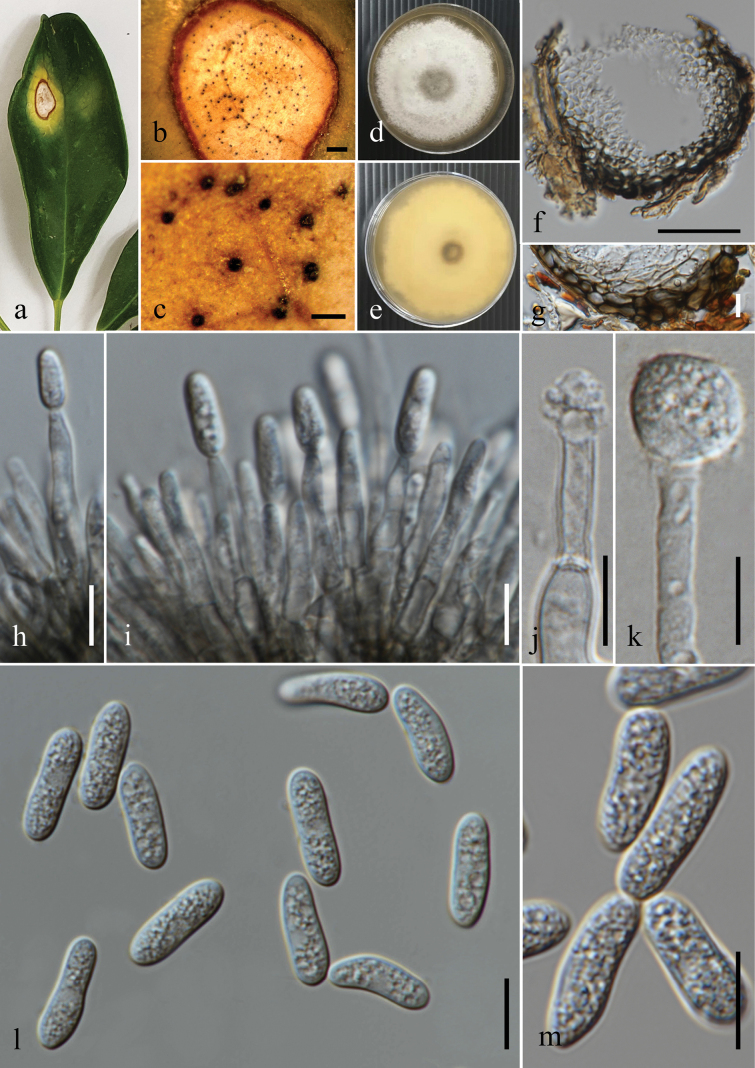
*Colletotrichumchrysophilum*. **a.** Leaf spot (*Terminalia* sp.); **b.** Close up of a leaf spot; **c.** Conidiomata on substrate; **d, e.** Top and reverse of colony on PDA after 3 d **f** Section through a conidioma; **g.** Conidiomatal wall; **h, i.** Conidiogenesis and developing conidia; **j, k.** Appressoria; **l, m.** Conidia. Scale bars: 1 mm (**b**); 200 μm (**c**); 50 μm (**f**); 10 μm (**g–m**).

###### Culture characteristics.

Colonies on PDA reaching approximately 45 mm diam. after 7 d of incubation at 25 °C; mycelium greyish white, elevation flat or raised, aerial and filamentous with an entire or undulate margin.

###### Specimen examined.

Thailand • Chiang Rai Province, around the vicinity of Central Plaza, associated with leaf spots of *Terminalia* sp. (Combretaceae), 11 Jul 2019, D. Gomdola DG01-SM (MFLU 25-0005), living culture MFLUCC 25-0006.

###### GenBank accession numbers.

ITS = PV263293; *GAPDH* = PV290899; *CHS*1 = PV274250; *ACT* = PV297876; *TUB*2 = PV295619; *H*3 = PV400141; and *CAM* = PV299285.

###### Known hosts, distributions, and lifestyles

**(listed chronologically). *Endophytic*** on *Theobromacacao* and *Genipaamericana* in Panama (Rojas et al. 2010) and *Terpsichoretaxifolia* in Puerto Rico (Doyle et al. 2013).

***Pathogenic*; *anthracnose*** on fruits of *Musa* sp. in Minas Gerais, Pernambuco, Santa Catarina, and São Paulo states in Brazil and Mexico (Vieira et al. 2017; Fuentes-Aragón et al. 2021), leaves of *Anacardiumhumile* and *A.occidentale* in Brazil (Veloso et al. 2018, 2021), fruits of *Mangiferaindica* in Mexico (Fuentes-Aragón et al. 2020a), fruits of *Perseaamericana* in Mexico (Fuentes-Aragón et al. 2020b) and Brazil (Soares et al. 2021), leaves of *Bauhiniaforficata* in Brazil (de Souza Junior et al. 2021), leaves of *Manihotesculenta* in Brazil (Machado et al. 2021), fruits of *Caricapapaya* in Mexico (Pacheco-Esteva et al. 2022), and leaves of V*accinium corymbosum* in Brazil (Soares et al. 2022).

***Bitter rot*** of *Malusdomestica* in New York (Khodadadi et al. 2020) and Spain (Cabrefiga et al. 2022).

***Preharvest decay*** of *Malusdomestica* in Italy (Deltedesco and Oettl 2023).

***Leaf spots*** on *Euterpeoleracea* and *Malusdomestica* in Brazil and Uruguay (Astolfi et al. 2022; dos Santos et al. 2022; Andrello et al. 2024).

Associated with leaf spots of *Terminalia* sp. in Thailand (this study).

###### Notes.

Our isolate (MFLUCC 25-0006) grouped with *Colletotrichumchrysophilum* (URM7368, A20_F13_004, PP_212b, PP_211a, PP_209c, PP_208d, PP_210d, and CBS 146410) with 100% ML and 1.00 PP support (Fig. 2). Based on our phylogenetic analyses, *C.chrysophilum* is positioned within the *C.gloeosporioides* species complex (Figs 1, 2), congruent with the findings of Vieira et al. (2017). No nucleotide difference was observed in ITS (495 bp), *GAPDH* (219 bp), *CHS*1 (267 bp), *ACT* (216 bp), *TUB*2 (648 bp), and *CAM* (656 bp) regions between our isolate (MFLUCC 25-0006) and *C.chrysophilum* (URM7368).

MFLUCC 25-0006 is morphologically similar to the ex-type of *C.chrysophilum* (URM7368), producing hyaline, smooth-walled, guttulate, aseptate, and cylindrical or oblong conidia with rounded ends (Vieira et al. 2017). The conidial L/W ratio of our isolate is similar to that of *C.chrysophilum* (URM7368) (L/W ratio = 3.1 vs. 3.1).

Based on phylogenetic and morphological species concepts, we identify our isolate as *Colletotrichumchrysophilum*. This study represents the first report of *C.chrysophilum* associated with leaf spots on *Terminalia* sp. and establishes a new geographical record in Thailand.

##### ﻿ Colletotrichum endophyticum

Taxon classificationFungiGlomerellalesGlomerellaceae

Manamgoda, Udayanga, L. Cai & K.D. Hyde [as ‘ endophytica’], in Manamgoda et al. Fungal Diversity 61:110 (2013)

7F45328E-D232-5359-98B7-0768AA3C6D95

Index Fungorum: IF565248

Facesoffungi Number: FoF14040

Figs 2 , 8

###### Description.

Associated with leaf spots, blight, and blotches. ***Leaf spots*** circular, brown, surrounded with a dark brown to black margin. ***Leaf blight*** and ***leaf blotches*** brown to dark brown. Sexual morph on substrate: ***Ascomata*** 100–150 × 100–120 µm (x̄ = 122 × 112 µm, n = 10), solitary or aggregated, semi-immersed, globose to subglobose, black, creamy at the center, ostiolate. ***Setae*** not observed. ***Peridium*** 9.5–18.5 µm thick (x̄ = 15.5 µm, n = 10), composed of 2–3 layers of thick-walled pseudoparenchymatous cells of ***textura angularis*. *Asci*** 54–88 × 12–21 µm (x̄ = 68.6 × 17.3 µm, n = 10), operculate, unitunicate, cylindrical to truncate or obovoid, 6–8-spored. ***Ascospores*** 15–17 × 4.5–6 µm (x̄ = 15.5 × 5.1 µm, n = 20; L/W ratio = 3.0), uniseriate or biseriate, hyaline, cylindrical to subellipsoidal or oblong, smooth-walled, guttulate, aseptate, mostly with rounded or obtuse ends. Asexual morph on substrate: ***Conidiomata*** 120–250 × 120–150 µm (x̄ = 200 × 140 µm, n = 10), semi-immersed, scattered, globose to subglobose, black, sometimes erumpent, exuding creamy orange conidial mass. ***Setae*** not observed. Asexual morph on PDA: ***Conidiomata*** 200–1000 µm diam. (x̄ = 400 µm, n = 10), semi-immersed, scattered or segregated, globose to subglobose, exuding orange conidial mass. ***Setae*** not observed. ***Conidiophores*** 20–25 µm long (x̄ = 21.6 µm, n = 10), formed directly from mycelium, hyaline, cylindrical, branched, or unbranched. ***Conidiogenous cells*** 6–9 × 3–4.5 µm (x̄ = 7 × 3.5 µm, n = 10), hyaline, cylindrical, or ampulliform, straight or flexuous, tapering towards the apex. ***Conidia*** 14–19 × 4–6 µm (x̄ = 15.9 × 4.9 µm, n = 30; L/W ratio = 3.2), hyaline, cylindrical to ovoid, smooth-walled, guttulate, aseptate, with rounded ends. ***Appressoria*** 8–11 × 7–8 µm (x̄ = 8.5 × 7.5 µm, n = 5), hyaline, single-celled, irregular, smooth-walled or verruculose.

**Figure 8.**
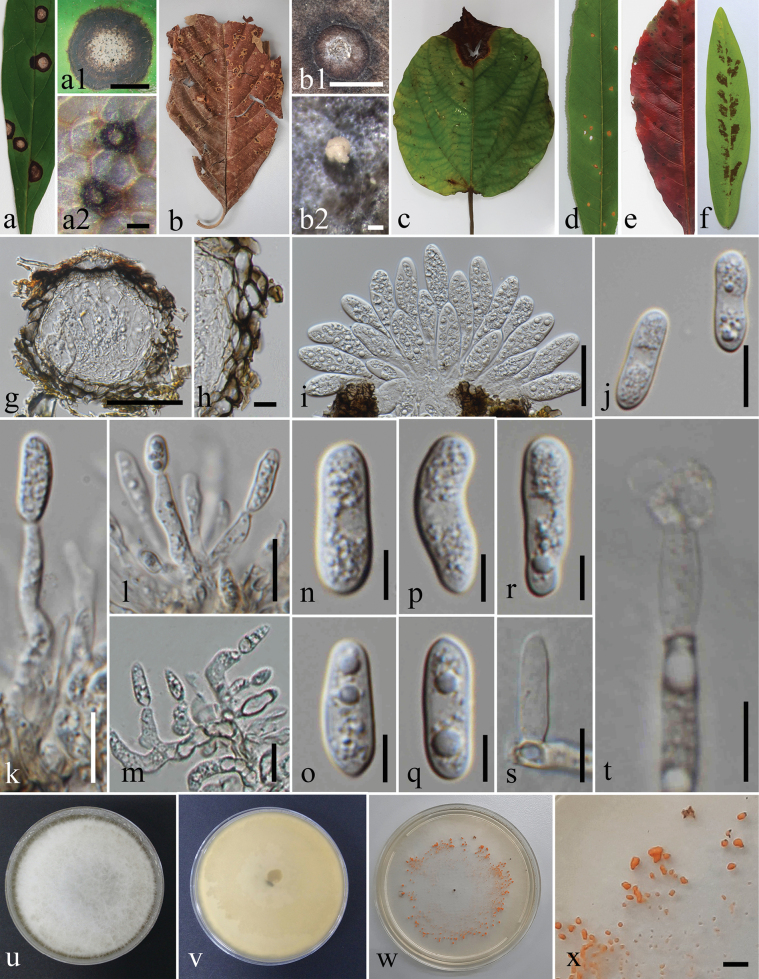
*Colletotrichumendophyticum*. **a, a1, a2.***Schefflera* sp. **a.** Diseased leaf with spots; **a1.** Close up of a leaf spot; **a2.** Ascomata on substrate; **b, b1, b2.***Artocarpusheterophyllus*; **b.** Fallen dried leaf with spots; **b1.** Close up of a leaf spot; **b2.** Conidioma sporulating on substrate; **c.** Leaf blight (*Ficusauriculata*); **d.** Leaf spots (*Begonialuxurians*); **e.** Fallen dried leaf with spots (*Castanopsis* sp.); **f.** Leaf blotches (*Cassia* sp.); **g.** Section through an ascoma; **h.** Peridium; **i.** Asci; **j.** Ascospores; **k–m.** Conidiogenesis and developing conidia; **n–r.** Conidia; **s.** Germinated conidium; **t.** Appressorium formed on PDA; **u, v.** Top and reverse of colony on PDA after 7 d; **w, x.** Spore mass formation on PDA after 21 d. Scale bars: 3 mm (**a1, b1**); 50 μm (**a2, b2, g, I, x**); 10 μm (**h, j–m, s, t**); 5 μm (**n–r**).

###### Culture characteristics.

Colonies on PDA reaching approximately 70 mm diam. after 7 d of incubation at 25 °C; mycelium initially white, becoming greyish white to dark grey at the center with age, elevation flat or raised, aerial and dense, with an entire margin, producing orange conidial mass.

###### Specimens examined.

Thailand • Chiang Mai Province, around vicinity of Mushroom Research Center, associated with leaf spots of *Schefflera* sp. (Araliaceae), 7 Jul 2021, D. Gomdola Div15-L1 (MFLU 25-0006), living culture MFLUCC 25-0007; Thailand • Chiang Mai Province, around vicinity of Mushroom Research Center, associated with spots on dried dead leaves of *Artocarpusheterophyllus* (Moraceae), 4 Apr 2023, D. Gomdola F6-A (MFLU 25-0007), living culture MFLUCC 25-0008; Thailand • Chiang Mai Province, Doi Inthanon National Park, Highland Fisheries Unit, associated with leaf blight of *Ficusauriculata* (Moraceae), 18 Oct 2021, D. Gomdola DGM3(L1)-A (MFLU 25-0008, BHH 50481), living culture MFLUCC 25-0009; Thailand • Chiang Mai Province, Mae On District, associated with leaf spots of *Begonialuxurians* (Begoniaceae), 26 Jun 2020, D. Gomdola Div10(L3)-T (MFLU 25-0009), living culture MFLUCC 25-0010; Thailand • Tak Province, associated with spots on dried dead leaves of *Castanopsis* sp. (Fagaceae), 16 Oct 2019, D. Gomdola DG153-T2 (MFLU 25-0010), living culture MFLUCC 25-0011; Thailand • Chiang Mai Province, Mae On District, associated with leaf blotches of *Cassia* sp. (Fabaceae), 25 Jun 2020, D. Gomdola Div31(T)-A (MFLU 25-0011), living culture MFLUCC 25-0012.

###### GenBank accession numbers.

MFLUCC 25-0007; ITS = PV263294; *GAPDH* = PV290900; *CHS*1 = PV274251; *ACT* = PV297877; and *TUB*2 = PV295620; MFLUCC 25-0008; ITS = PV263295; *GAPDH* = PV290901; *CHS*1 = PV274252; *ACT* = PV297878; *TUB*2 = PV295621; *H*3 = PV400142; and *CAM* = PV299286; MFLUCC 25-0009; ITS = PV263296; *GAPDH* = PV290902; *CHS*1 = PV274253; and *ACT* = PV297879; MFLUCC 25-0010; ITS = PV263297; *GAPDH* = PV290903; *CHS*1 = PV274254; *ACT* = PV297880; *TUB*2 = PV295622; *H*3 = PV400143; and *CAM* = PV299287; MFLUCC 25-0011; ITS = PV263298; *GAPDH* = PV290904; *CHS*1 = PV274255; *ACT* = PV297881; *TUB*2 = PV295623; *H*3 = PV400144; and *CAM* = PV299288; and MFLUCC 25-0012; ITS = PV263299; *GAPDH* = PV290905; *CHS*1 = PV274256; *ACT* = PV297882; *TUB*2 = PV295624; *H*3 = PV400145; and *CAM* = PV299289.

###### Known hosts, distributions, and lifestyles

**(listed chronologically). *Endophytic*** on leaves of *Pennisetumpurpureum* in Thailand (Manamgoda et al. 2013) and fruits and leaves of *Capsicumannuum* in China (Diao et al. 2017).

***Saprobic*** on an unknown wild fruit in Thailand (Udayanga et al. 2013).

***Pathogenic*; *anthracnose*** on leaves of *Camelliasinensis* in China (Wang et al. 2016a), leaves and berries of Coffeacanephoravar.robusta in China (Cao et al. 2019b), fruits and leaves of *Perseaamericana* in Sri Lanka (Dissanayake et al. 2021) and Thailand (Armand and Jayawardena 2024), and leaves of *Philodendronbipinnatifidum* in China (Zhang et al. 2023c).

***Fruit lesion*** of *Capsicumannuum* in Thailand (de Silva et al. 2019).

Leaf spots of *Acaciaconfusa* and *Bauhiniablakeana* in China (Manawasinghe et al. 2022; Liang et al. 2023; Zhang et al. 2023b).

Associated with anthracnose of fruits and leaves of *Mangiferaindica* (Li et al. 2019) and leaf spots of *Artocarpusheterophyllus*, *Begonialuxurians*, *Castanopsis* sp., and *Schefflera* sp., leaf blight of *Ficusauriculata*, and leaf blotches of *Cassia* sp. in Thailand (this study).

###### Notes.

Our isolates (MFLUCC 25-0007, MFLUCC 25-0008, MFLUCC 25-0009, MFLUCC 25-0010, MFLUCC 25-0011, and MFLUCC 25-0012) grouped with other strains of *Colletotrichumendophyticum* with 100% ML and 1.00 PP support (Fig. 2). In our phylogenetic analyses, *C.endophyticum* is positioned within the *C.gloeosporioides* species complex (Figs 1, 2), consistent with findings of Manamgoda et al. (2013), Jayawardena et al. (2016a), and Zhang et al. (2023b). The intraspecies nucleotide differences between our isolates and the ex-type of *C.endophyticum* (MFLUCC 13-0418) are given in Table 1.

**Table 1.** Intra-species nucleotide differences between our isolates and the ex-type of *C.endophyticum* (MFLUCC 13-0418). The numbers in each cell represent the number of nucleotide differences relative to the total length of the corresponding gene region.

Gene regions	MFLUCC 25-0007	MFLUCC 25-0008	MFLUCC 25-0011	MFLUCC 25-0009	MFLUCC 25-0010	MFLUCC 25-0012
ITS	1/476	1/476	4/476	1/476	4/476	4/476
*GAPDH*	4/227	8/261	1/228	4/249	5/258	8/240
*CHS*1	2/247	3/279	4/279	5/245	1/279	5/277
*ACT*	1/234	2/220	2/236	3/234	2/235	2/234
*TUB*2	0/668	8/668	0/656	N/A	0/668	10/668
*H*3	N/A	6/360	0/360	N/A	1/360	7/360

N/A: Not applicable.

Our isolates morphologically resemble the ex-type of *C.endophyticum* (MFLUCC 13-0418), having hyaline, cylindrical, or ampulliform conidiogenous cells and hyaline, smooth-walled, guttulate, aseptate, and cylindrical to ovoid conidia with rounded ends. They also share similar colony characteristics, observed as white to greyish mycelium (Manamgoda et al. 2013; Zhang et al. 2023b). Additionally, the conidial L/W ratio of our isolates is similar to that of *C.endophyticum* (MFLUCC 13-0418) (L/W ratio = 3.2 vs. 3.4).

Based on phylogenetic and morphological species concepts, we identify our isolates as *Colletotrichumendophyticum*. This study represents six new host records for *C.endophyticum* associated with leaf spots of *Begonialuxurians* and *Schefflera* sp., spots on dried dead leaves of *Castanopsis* sp. and *Artocarpusheterophyllus*, leaf blight of *Ficusauriculata*, and leaf blotches of *Cassia* sp. in Thailand. Additionally, this is the first report for its sexual morph.

##### ﻿ Colletotrichum fructicola

Taxon classificationFungiGlomerellalesGlomerellaceae

Prihast., L. Cai & K.D. Hyde, in Prihastuti et al. Fungal Diversity 39:96 (2009)

04E751BE-D2AB-529A-A537-A3B64F10ED19

Index Fungorum: IF515409

Facesoffungi Number: FoF06767

Figs 2 , 9

###### Description.

Associated with leaf spots, blight, and blotches. ***Leaf spots*** circular or irregular, pale brown to brown, surrounded with a dark brown margin. Leaf blight brown, surrounded with a dark brown margin. ***Leaf blotches*** reddish brown to dark brown. Sexual morph on substrate: ***Ascomata*** 75–150 × 80–150 µm (x̄ = 95 × 98 µm, n = 5), solitary, semi-immersed, globose to subglobose, brown, ostiolate. ***Setae*** not observed. Sexual morph on PDA: ***Ascomata*** 100–200 × 100–190 µm (x̄ = 131 × 123 µm, n = 5), solitary or aggregated, semi-immersed or superficial, globose to subglobose, black. ***Setae*** not observed. ***Peridium*** 8–38 µm thick (x̄ = 19.1 µm, n = 10), composed of 3–4 layers of thick-walled pseudoparenchymatous cells of ***textura angularis*. *Asci*** 55–70 × 8–11 µm (x̄ = 63 × 9.5 µm, n = 10), operculate, unitunicate, cylindrical to clavate or cymbiform, 6–8-spored. ***Ascospores*** 13–23.5 × 4–6.5 µm (x̄ = 17.9 × 5.1 µm, n = 25; L/W ratio = 3.5), uniseriate or biseriate, hyaline, subellipsoidal or oblong, reniform to falcate, somewhat fusiform, slightly curved, smooth-walled, guttulate, aseptate, mostly with obtuse or acute ends. Asexual morph on substrate: ***Conidiomata*** 80–150 × 60–140 µm (x̄ = 100 × 90 µm, n = 5), solitary, semi-immersed, globose to subglobose, brown, producing creamy to orange conidial mass. ***Setae*** not observed. Asexual morph on PDA: ***Conidiomata*** 200–800 µm diam. (x̄ = 350 µm, n = 10), semi-immersed, scattered or aggregated, globose to subglobose, exuding creamy to orange conidial mass. ***Setae*** not observed. ***Conidiophores*** formed directly from mycelium, hyaline, cylindrical, branched, or unbranched. ***Conidiogenous cells*** 6.5–22 × 2–4 µm (x̄ = 12.1 × 3 µm, n = 10), hyaline, cylindrical, or ampulliform, straight or flexuous, tapering towards the apex. ***Conidia*** 12–18 × 4.5–5.5 µm (x̄ = 15.4 × 5 µm, n = 25; L/W ratio = 3.1), hyaline, cylindrical to ovoid, smooth-walled, guttulate, aseptate, with rounded ends. ***Chlamydospores*** 6–7 × 6.5–8 µm (x̄ = 6.3 × 7.2 µm, n = 5), globose to subglobose, pale brown. ***Appressoria*** Not observed.

###### Culture characteristics.

Colonies on PDA reaching approximately 80 mm diam. after 7 d of incubation at 25 °C; mycelium initially white, becoming dark grey at the center when aged, elevation flat or raised, aerial and dense, with an entire margin.

**Figure 9.**
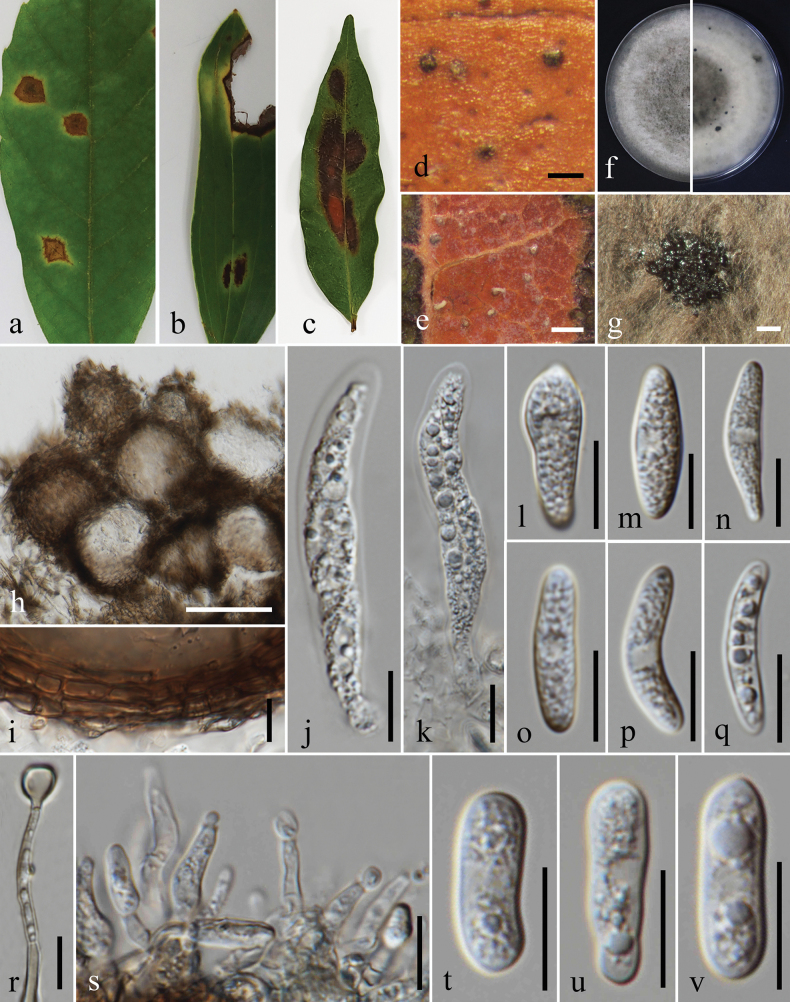
*Colletotrichumfructicola*. **a.** Leaf spots (*Castanea* sp.); **b.** Leaf blight (*Hedychium* sp.); **c.** Leaf blotches (*Rhododendron* sp.); **d.** Ascomata on substrate (*Rhododendron* sp.); **e.** Conidiomata sporulating on substrate (*Hedychium* sp.); **f.** Top and reverse of colony on PDA after 21 d; **g.** Clusters of ascomata formed on culture; **h.** Section through ascomata; **i.** Peridium; **j, k.** Asci; **l–q.** Ascospores; **r.** Chlamydospore; **s.** Conidiogenesis and developing conidia; **t–v.** Conidia. Scale bars: 500 μm (**d, e, g**); 100 μm (**h**); 10 μm (**i–v**).

###### Specimens examined.

Thailand • Chiang Mai Province, Omkoi District, Yiang Piang Subdistrict, associated with leaf spots of *Castanea* sp. (Fagaceae), 16 Oct 2019, D. Gomdola DG367-L2 (MFLU 25-0012), living culture MFLUCC 25-0013; DG367(L2)-A (MFLU 25-0013), living culture MFLUCC 25-0014; DG367(L2)-B (MFLU 25-0014), living culture MFLUCC 25-0015; Thailand • Chiang Mai Province, Doi Lo District, associated with leaf blight of *Hedychium* sp. (Zingiberaceae), 15 Oct 2019, D. Gomdola DG327 (MFLU 25-0015), living culture MFLUCC 25-0016; Thailand • Chiang Rai Province, around the vicinity of Central Plaza, associated with leaf blotches of *Rhododendron* sp. (Ericaceae), 11 Jul 2019, D. Gomdola DG03.1 (MFLU 25-0016), living culture MFLUCC 25-0017.

###### GenBank accession numbers.

MFLUCC 25-0013; ITS = PV263300; *GAPDH* = PV290906; *CHS*1 = PV274257; *ACT* = PV297883; and *TUB*2 = PV295625; MFLUCC 25-0014; ITS = PV263301; *GAPDH* = PV290907; *CHS*1 = PV274258; *ACT* = PV297884; and *H*3 = PV549703; MFLUCC 25-0015; ITS = PV263302; *GAPDH* = PV290908; *CHS*1 = PV274259; *ACT* = PV297885; and *H*3 = PV400146; MFLUCC 25-0016; ITS = PV263303; *GAPDH* = PV290909; *CHS*1 = PV274260; and *H*3 = PV400147; and *CAM* = PV299290; and MFLUCC 25-0017; ITS = PV263304; *GAPDH* = PV290910; *CHS*1 = PV274261; *ACT* = PV297886; *TUB*2 = PV295626; *H*3 = PV400148; and *CAM* = PV299291.

###### Known hosts, distributions, and lifestyles

**(listed chronologically). *Pathogenic on plants*; *Leaf spots*** of *Ficusedulis* in Germany and *Limonium* spp. in Israel (Weir et al. 2012); *Pyruspyrifolia* (Zhang et al. 2015), *Dalbergiahupeana* (Zhou et al. 2022), *Myricarubra* (Li et al. 2022a), *Ziziphusmauritiana* (Shu et al. 2021), *Zamiafurfuracea* (Manawasinghe et al. 2022), Liriodendronchinense×tulipifera (Wan et al. 2022), *Magnoliawufengensis* (Yin et al. 2022), *Illiciumverum* (Zhao et al. 2022), *Camelliasinensis*, *Curcumaphaeocaulis*, *Ilexchinensis*, *Ligustrumlucidum* and *Zingiberofficinale* (Zhang et al. 2023a), and *Celosiacristata*, *Cymbidiumsinense* and *Dendrobiumnobile* in China (Zhang et al. 2023b); *Malusdomestica* in Uruguay (Casanova et al. 2017; Alaniz et al. 2019); and *Nephrolepiscordifolia* (Seifollahi et al. 2023) and *Rhizophoraapiculata* in Thailand (Norphanphoun and Hyde 2023).

***Leaf blotch*** of *Aesculuschinensis* in China (Sun et al. 2020) and brown blight of *Camelliasinensis* in Taiwan (Lin et al. 2023a).

***Shot-hole*** on leaves of *Prunussibirica* in China (Han et al. 2023).

***Brown sunken cladode spots*** of *Nopaleacochenillifera* in Brazil (Conforto et al. 2017).

***Anthracnose*** of *Dioscorea* spp. in Nigeria (Weir et al. 2012); *Pyrusbretschneideri*, *P.communis* and *P.pyrifolia* in China (Weir et al. 2012; Li et al. 2013; Fu et al. 2019) and *Pyruspyrifolia* × *P.communis* in Korea (Choi and Park 2021); *Citrus* spp. in China (Huang et al. 2013; Hu et al. 2019) and Iran (Arzanlou et al. 2015; Taheri et al. 2016); *Hylocerousundatus* and *Ziziphus* sp. in Thailand (Udayanga et al. 2013); *Mangiferaindica* in Brazil (Lima et al. 2013, 2015), India (Sharma et al. 2013), Korea (Joa et al. 2016), China (Li et al. 2019), Mexico (Tovar-Pedraza et al. 2020), Egypt (Ismail and El-Ganainy 2022) and Taiwan (Wu et al. 2020; Lin et al. 2023b); *Rubusglaucus* in Colombia (Afanador-Kafuri et al. 2014); *Gleditsiacaspica* in Iran (Arzanlou et al. 2015); *Prunuspersica* in USA (Hu et al. 2015), Korea (Lee et al. 2020) and China (Tan et al. 2022); *Camelliasinensis* in China (Liu et al. 2015; Wang et al. 2016a; Lu et al. 2018; Shi et al. 2018) and Indonesia (Weir et al. 2012; Liu et al. 2015); *Corchoruscapsularis* (Niu et al. 2016a, 2016b) and Fragaria×ananassa (Han et al. 2016; Jayawardena et al. 2016b; He et al. 2019; Chen et al. 2020; Jian et al. 2021); *Aucubajaponica* in China (Li et al. 2016) and Korea (Hassan et al. 2023); *Annona* spp. in Brazil (Costa et al. 2016, 2019); *Capsicum* spp. in China (Liu et al. 2016; Diao et al. 2017), Thailand (de Silva et al. 2019) and Malaysia (Noor and Zakaria 2018); *Nicotianatabacum* in China (Wang et al. 2016b); *Caricapapaya* in India (Saini et al. 2016), Mexico (Marquez-Zequera et al. 2018), Costa Rica (Ruiz-Campos et al. 2022) and Brazil (Vieira et al. 2022); *Fatsiajaponica* in china (Shi et al. 2017); *Malusdomestica* in Iran (Arzanlou et al. 2015) and Korea (Kim et al. 2018, 2020); *Anacardiumoccidentale*, *A.othonianum* and *A.humile* in Brazil (Veloso et al. 2018, 2021); *Juglansregia* (Wang et al. 2018; Li et al. 2023a) and *Pouteriacampechiana* in China (Yang et al. 2021); *Diospyroskaki* in Brazil (Carraro et al. 2019), Philippines (Evallo et al. 2022) and China (Zhang et al. 2023d); *Heveabrasiliensis* in China (Cao et al. 2019a) and Brazil (Santos de Oliveira et al. 2020); *Coffeaarabica* in China (Cao et al. 2019b) and Puerto Rico (Serrato-Diaz et al. 2020); *Salviagreggii* in Italy (Guarnaccia et al. 2019); *Vitislabruscana* and *V.vinifera* in Korea (Lim et al. 2020); *Manihotesculenta* in China (Liu et al. 2018) and Brazil (Bragança et al. 2016; Santos de Oliveira et al. 2020); *Dendrobiumofficinale* in China (Ma et al. 2019); *Cattleya* spp. and *Phalaenopsis* sp. in Brazil (Silva-Cabral et al. 2019); *Arecacatechu* (Cao et al. 2020), *Peucedanumpraeruptorum* (Ma et al. 2020), *Crinumasiaticum* (Qing et al. 2020), *Camelliaoleifera* (Wang et al. 2020) and Parispolyphyllavar.chinensis in China (Zhou et al. 2020); *Ceanothusthyrsiflorus*, *Hydrangeapaniculata*, *Cyclamenpersicum* and *Liquidambarstyraciflua* in Italy (Guarnaccia et al. 2021); *Perseaamericana* in Colombia (Gañán et al. 2015), Israel (Sharma et al. 2017), Mexico (Fuentes-Aragón et al. 2018), New Zealand (Hofer et al. 2021) and Thailand (Armand and Jayawardena 2024); *Alliumcepa* in Brazil (Henrique Lopes et al. 2021); *Musa* spp. (Huang et al. 2021a), *Eichhorniacrassipes* (Huang et al. 2021b) and *Eriobotryajaponica* in China (Kuang et al. 2021); *Camelliasinensis* in Taiwan (Lin et al. 2021); *Eucalyptus* spp. in South Africa (Mangwende et al. 2021); *Amomumvillosum* (Song et al. 2021), *Rubuscorchorifolius* (Wu et al. 2021), *Camelliachrysantha* (Zhao et al. 2021), *Cyclocaryapaliurus* (Zheng et al. 2021) and *Camelliagrijsii* (=*C.yuhsienensis*) in China (Chen et al. 2022); *Ziziphusjujuba* (=*Z.mauritiana*) in Taiwan (Duan and Chen 2022); *Atractylodesovata* in Korea (Hassan et al. 2022); *Cunninghamialanceolata* (He et al. 2022), *Prunussalicina* (Huang et al. 2022a) and *Phoebesheareri* in China (Huang et al. 2022b); *Actinidia* spp. in China and Japan (Huang et al. 2022c; Poti et al. 2023); *Caryaillinoinensis* (Chang et al. 2022), *Macadamiaintegrifolia* (Li et al. 2023b), *Luffacylindrica* (Li et al. 2022b), *Loropetalumchinense* (Qiu et al. 2022), *Prunusavium* (Tang et al. 2022), *Bletillastriata* (Wang et al. 2022), *Brassicaparachinensis* (Yu et al. 2022a), *Radermacherasinica* (Yu et al. 2022b), *Arachishypogaea* (Gong et al. 2023), *Osmanthusfragrans* (He et al. 2023; Sui et al. 2024), *Averrhoacarambola* (Li and Zhang 2023), *Caryacathayensis* (Ma et al. 2023), *Tetrapanaxpapyrifer* (Tang et al. 2023) and *Glycinemax* in China (Xu et al. 2023).

***Fruit rot*** of *Perseaamericana* in Australia (Weir et al. 2012), *Nepheliumlappaceum* in Puerto Rico (Serrato-Diaz et al. 2017), and *Ziziphusmauritiana* in China (Fan et al. 2022).

***Ripe rot*** of *Vitis* spp. in Brazil (Echeverrigaray et al. 2020).

***Bitter rot*** of *Malusdomestica* in China (Fu et al. 2013), the USA (Weir et al. 2012; Munir et al. 2016), Brazil (Weir et al. 2012; Velho et al. 2015, 2018, 2019; Moreira et al. 2019), Uruguay (Alaniz et al. 2015; Velho et al. 2015), Japan (Yokosawa et al. 2017), Korea (Oo et al. 2018; Park et al. 2018), France (Nodet et al. 2019), and Italy (Wenneker et al. 2021).

**Associated with** spathe rot, spadix rot, and leaf spots of *Anthuriumandraeanum* in Sri Lanka (Vithanage et al. 2021); leaf spots of *Castanea* sp., leaf blight of *Hedychium* sp., and leaf blotches of *Rhododendron* sp. in Thailand (this study).

*Colletotrichumfructicola* was also reported from Fragaria×ananassa in Canada and the USA (Weir et al. 2012) and *Morusalba* in China but showed no pathogenicity (Xue et al. 2019). Furthermore, it was reported to cause diseases on *Verniciamontana*, *Cinnamomumcamphora*, *Paulowniafortunei*, and *Schimasuperba* in China (Sui et al. 2024).

***Pathogenic on a nematode*** in China; infects horsehair worms (*Chordodesformosanus*), a parasite of praying mantises (De Vivo et al. 2021).

***Pathogenic on humans***; causes *Colletotrichum* keratitis, a fungal infection of human eyes (Hung et al. 2020).

***Endophytic*** on *Tetragastrispanamensis* and *Theobromacacao* in Panama (Weir et al. 2012), *Cymbopogoncitratus* and *Pennisetumpurpureum* in Thailand (Manamgoda et al. 2013), *Licaniatomentosa* in Brazil (Lisboa et al. 2018), *Dendrobium* spp. in China (Ma et al. 2018), *Coffeaarabica* in Thailand (Numponsak et al. 2018), and *Magnoliacandolli* in China (De Silva et al. 2021).

###### Notes.

Our isolates (MFLUCC 25-0013, MFLUCC 25-0014, MFLUCC 25-0015, MFLUCC 25-0016, and MFLUCC 25-0017) grouped with other strains of *Colletotrichumfructicola* with 99% ML and 1.00 PP support (Fig. 2). *Colletotrichumfructicola* is located in the *C.gloeosporioides* species complex (Figs 1, 2), consistent with findings of Prihastuti et al. (2009), Ma et al. (2018), Norphanphoun and Hyde (2023), and Zhang et al. (2023b). No intraspecies nucleotide differences were observed between our isolates and the ex-type of *C.fructicola* (ICMP 18581) across the ITS, *GAPDH*, *CHS*1, *ACT*, and *TUB*2 regions. However, a sequence divergence of 0.7% (5/731 bp) was observed in *CAM* between our isolate (MFLUCC 25-0017) and *C.fructicola* (ICMP 18581).

Our isolates morphologically resemble the ex-type of *C.fructicola* (ICMP 18581), having hyaline, smooth-walled, guttulate, and aseptate conidia and ascospores, with the conidia being cylindrical to ovoid with rounded ends and ascospores being oblong, reniform to falcate with obtuse or acute ends (Prihastuti et al. 2009). Notably, the ascospore and conidial lengths of our isolates vary slightly with other strains of *C.fructicola*. However, the L/W ratios of our isolates are similar to those of other *C.fructicola* strains. The ascospore L/W ratio of our isolates is 3.5, while those from other studies are ICMP 18581 = 3.6 (Prihastuti et al. 2009), MFLUCC 14-0087 = 3.4 (Ma et al. 2018), MFLUCC 17-1752 = 3.2 (Norphanphoun and Hyde 2023), and ZHKUCC 23-0829 = 3.7 (Zhang et al. 2023b). The conidial L/W ratio of our isolates is 3.1, while those from other studies are ICMP 18581 = 3.2 (Prihastuti et al. 2009), MFLUCC 14-0087 = 2.9 (Ma et al. 2018), and MFLUCC 17-1752 = 2.6 (Norphanphoun and Hyde 2023).

Based on phylogenetic and morphological species concepts, we identify our isolates as *Colletotrichumfructicola*. This study represents three new host records for *C.fructicola* associated with leaf spots of *Castanea* sp., leaf blight of *Hedychium* sp., and leaf blotches of *Rhododendron* sp. in Thailand.

##### ﻿ Colletotrichum jiangxiense

Taxon classificationFungiGlomerellalesGlomerellaceae

F. Liu & L. Cai, in Liu et al. Persoonia 35:82 (2015)

559EBA01-69CD-5592-81E2-857889F78E10

Index Fungorum: IF809161

Facesoffungi Number: FoF17277

Figs 2 , 10

###### Description.

Associated with leaf spots of *Artocarpus* sp. ***Leaf spots*** circular, oval, or irregular, pale brown to brown, surrounded with a dark brown margin. Sexual morph: Not observed. Asexual morph on substrate: ***Conidiomata*** 200–500 × 150–400 µm (x̄ = 300 × 200 µm, n = 10), acervular, semi-immersed, scattered or gregarious, globose to subglobose, dark brown to black, erumpent, exuding creamy orange conidial mass. ***Setae*** not observed. Asexual morph on PDA: ***Conidiomata*** 200–1000 µm diam. (x̄ = 400 µm, n = 10), semi-immersed, scattered or gregarious, globose to subglobose, dark brown to black, exuding creamy orange conidial mass. ***Setae*** not observed. ***Conidiophores*** hyaline, sometimes branched. ***Conidiogenous cells*** 12–19 × 2–5 µm (x̄ = 14.9 × 3.2 µm, n = 10), hyaline, cylindrical to ampulliform, straight or flexuous. ***Conidia*** 14–21 × 4–6.5 µm (x̄ = 16.5 × 5.2 µm, n = 30; L/W ratio = 3.2), hyaline, cylindrical to ellipsoidal, smooth-walled, guttulate, aseptate, with rounded ends (sometimes tapering towards one end), forming conidial anastomosis tubes. ***Conidial anastomosis tubes*** 2–16 × 1–2 µm (x̄ = 8.2 × 1.5 µm, n = 10), hyaline, smooth-walled, aseptate. ***Appressoria*** not observed.

###### Culture characteristics.

Colonies on PDA reaching approximately 55 mm diam. after 7 d of incubation at 25 °C; mycelium white to grey, elevation flat, cottony, with an entire margin.

**Figure 10.**
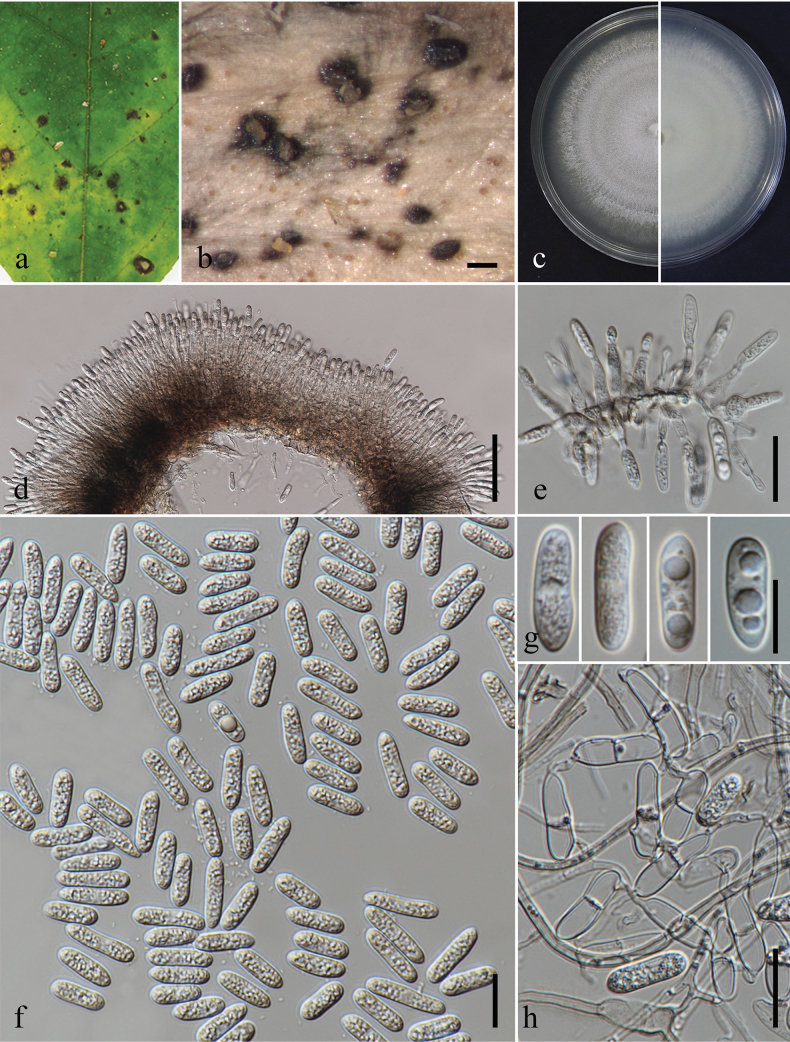
*Colletotrichumjiangxiense*. **a.** Leaf spots (*Artocarpus* sp.); **b.** Conidiomata on substrate; **c.** Top and reverse of colony on PDA after 7 d; **d, e.** Conidiogenesis and developing conidia; **f, g.** Conidia; **h.** Conidia forming conidial anastomosis tubes. Scale bars: 200 μm (**b**); 50 μm (**d**); 20 μm (**e**); 10 μm (**f–h**).

###### Specimen examined.

Thailand • Chiang Mai: Omkoi District, Yiang Piang Subdistrict, associated with leaf spots of *Artocarpus* sp. (Moraceae), 16 Oct 2019, *D. Gomdola DG360* (MFLU 25-0017), living culture MFLUCC 25-0018.

###### GenBank.

ITS = PV263305; *TUB*2 = PV295627; *H*3 = PV400149.

###### Known hosts, distributions, and lifestyles

**(listed chronologically). *Endophytic*** on *Camelliasinensis* in China (Liu et al. 2015; Jayawardena et al. 2016a).

***Pathogenic*; *brown lesions*** on leaves of *Camelliasinensis* in China (Liu et al. 2015).

***Anthracnose*** on fruits of *Perseaamericana* in Mexico (Ayvar-Serna et al. 2020; Fuentes-Aragón et al. 2020b).

***Leaf spots*** of *Fraxinusamericana* in China (Chang et al. 2023).

Associated with leaf spots of *Artocarpus* sp. in Thailand (this study).

###### Notes.

Our isolate (MFLUCC 25-0018) grouped with *Colletotrichumjiangxiense* (CGMCC 3.1736, 22N642, SYD-9, and SYD-4) with 90% ML support (Fig. 2). However, this clade has low support for other species. Similar to the study conducted by Liu et al. (2015), this research shows that *C.jiangxiense* is located in the *C.gloeosporioides* species complex (Figs 1, 2). Nucleotide sequence comparisons between our isolate and *C.jiangxiense* (CGMCC 3.17363) showed no difference in the ITS (505 bp) and *TUB*2 (667 bp) regions.

MFLUCC 25-0018 morphologically resembles the ex-type of *C.jiangxiense* (CGMCC 3.17363), having hyaline, aseptate, smooth-walled, and cylindrical conidia (Liu et al. 2015). The conidial L/W ratio of our isolate is close to that of *C.jiangxiense* (CGMCC 3.17363) (L/W ratio = 2.9 vs. 3.2).

Based on phylogenetic and morphological species concepts, we identify our isolate as *Colletotrichumjiangxiense*. This study represents the first report of *C.jiangxiense* associated with leaf spots on *Artocarpus* sp. and establishes a new geographical record in Thailand.

#### ﻿*Colletotrichumspaethianum* species complex

##### ﻿ Colletotrichum dendrobii

Taxon classificationFungiGlomerellalesGlomerellaceae

Gomdola, K.D. Hyde & Jayaward. sp. nov.

1F17A4CD-5E56-5CEC-8A25-0E7083FE0119

Index Fungorum: IF901618

Facesoffungi Number: FoF17275

Figs 3 , 11

###### Holotype.

MFLU 25-0018.

###### Etymology.

The epithet refers to the host genus, *Dendrobium*, from which the species was isolated.

###### Description.

Associated with pod blight of *Dendrobium* sp. ***Pod blight*** elongated, pale brown to brown, surrounded with a dark brown margin. Sexual morph: Not observed. Asexual morph on substrate: ***Conidiomata*** 200–300 × 180–200 µm (x̄ = 240 × 190 µm, n = 5), acervular, semi-immersed, scattered or gregarious, dark brown to black. ***Setae*** 30–200 µm long (x̄ = 103 µm, n = 30), scattered or aggregated, straight or flexuous, smooth-walled, 1–5-septate, brown to dark brown, base darker and apex paler, base 3.5–12.5 µm wide (x̄ = 7.1 µm, n = 30), cylindrical or ampulliform, tapering to 1.5–5.5 µm (x̄ = 3.1 µm, n = 30) at the apex. Asexual morph on PDA: ***Conidiomata*** 200–1000 µm diam. (x̄ = 450 µm, n = 10), semi-immersed, scattered or aggregated, globose to subglobose, black, exuding orange conidial mass. ***Setae*** not observed. ***Conidiophores*** 13–30 µm long (x̄ = 19.7 µm, n = 10), hyaline, branched or unbranched, smooth-walled. ***Conidiogenous cells*** 6–14(–20) × 2.5–4.5 µm (x̄ = 8.3 × 3.5 µm, n = 10), hyaline, cylindrical to ampulliform, sometimes elongated, straight or flexuous. ***Conidia*** 11–19 × 4–6 µm (x̄ = 16.7 × 4.8 µm, n = 30; L/W ratio = 3.5), hyaline, falcate or fusiform, sometimes cylindrical or irregular in shape, smooth-walled, guttulate, aseptate, tapering towards both ends, base conical or rounded, apex acute. ***Appressoria*** 15–17 × 17–19 µm (x̄ = 15.6 × 17.8 µm, n = 5), hyaline, single-celled, globose to subglobose, smooth-walled or verruculose.

**Figure 11.**
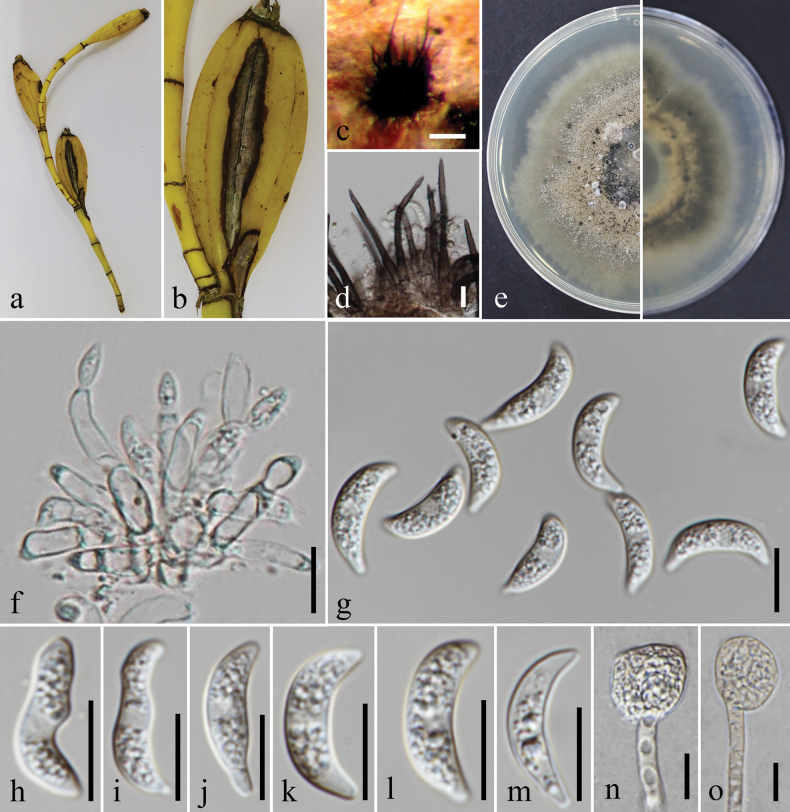
*Colletotrichumdendrobii* (MFLU 25-0018, holotype); **a, b.** Pod blight (*Dendrobium* sp.); **c.** Conidiomata with setae on substrate; **d.** Setae; **e.** Top and reverse of colony on PDA after 30 d; **f.** Conidiogenesis and developing conidia; **g–m.** Conidia; **n, o.** Appressoria. Scale bars: 200 μm (**c**); 10 μm (**d, f–o**).

###### Culture characteristics.

Colonies on PDA reaching approximately 70 mm diam. after 7 d of incubation at 25 °C; mycelium initially greyish white, becoming dark grey to olivaceous brown with age; elevation flat, with an entire or undulate margin.

###### Specimen examined.

Thailand • Chiang Mai Province, Omkoi District, Yiang Piang Subdistrict, associated with pod blight of *Dendrobium* sp. (Orchidaceae), 16 Oct 2019, D. Gomdola DG386 (MFLU 25-0018, **holotype**), ex-type MFLUCC 25-0019.

###### Additional specimen examined.

Thailand • Chiang Mai Province, Omkoi District, Yiang Piang Subdistrict, associated with pod blight of *Dendrobium* sp. (Orchidaceae), 17 Oct 2019, D. Gomdola DG387 (MFLU 25-0019), living culture MFLUCC 25-0020.

###### GenBank accession numbers.

MFLUCC 25-0019; ITS = PV263306; *GAPDH* = PV290911; *CHS*1 = PV274262; *ACT* = PV297887; *H*3 = PV400150; and *CAM* = PV299292; MFLUCC 25-0020; ITS = PV263307; and *GAPDH* = PV290912.

###### Known hosts, distributions, and lifestyles.

Associated with pod blight of *Dendrobium* sp. in Thailand (this study).

###### Notes.

Our isolates (MFLUCC 25-0019 and MFLUCC 25-0020) grouped with 100% ML and 1.00 PP support. This sub-clade forms a sister clade to *Colletotrichumverruculosum* (IMI 45525) with strong support (100% ML and 1.00 PP), indicating a close phylogenetic relationship (Fig. 3). In the phylogenetic analyses, our isolates are positioned within the *C.spaethianum* species complex (Figs 1, 3). The interspecies nucleotide sequence comparison between our isolates (MFLUCC 25-0019 and MFLUCC 25-0020) and *C.verruculosum* (IMI 45525) revealed the following divergence pattern: 1.3% in ITS (7/519 bp), 3.1% in *GAPDH* (6/196 bp), 1.9% in *ACT* (4/211 bp), and 2.4% in *H*3 (9/371 bp) regions, but no nucleotide differences in *CHS*1 (251 bp) between MFLUCC 25-0019 and *C.verruculosum*.

Morphologically, the conidiophores and conidiogenous cells of our isolates are hyaline, while those of *C.verruculosum* (IMI 45525) are pale brown. Additionally, conidiophores of our isolates are shorter than those of *C.verruculosum* (up to 30 µm vs. 110 µm long). Conidiogenous cells of our isolates are also smaller compared to those of *C.verruculosum* (6–14(–20) × 2.5–4.5 µm vs. 10–25 × 3–5 µm). Setae of our isolates are 1–5-septate and 30–200 µm long, while those of *C.verruculosum* are 2–4-septate and 70–160 µm long. The conidial L/W ratio of our isolates differs from that of *C.verruculosum* (L/W ratio = 3.5 vs. 4.6) (Damm et al. 2009).

Based on phylogenetic analyses and morphological data following recommendations proposed by Chethana et al. (2021) and Jayawardena et al. (2021b), we establish our isolates as a new species, *Colletotrichumdendrobii*, associated with pod blight of *Dendrobium* sp. in Thailand.

The primary feature distinguishing *Colletotrichumdendrobii* from *C.verruculosum* (IMI 45525) is its variable and irregular conidial shape, along with their distinct phylogenetic lineages.

#### ﻿*Colletotrichum* singleton species

##### ﻿ Colletotrichum musichiangmaiense

Taxon classificationFungiGlomerellalesGlomerellaceae

Gomdola, K.D. Hyde & Jayaward. sp. nov.

D3AAFB38-01A3-5897-A148-F4BAA978DD19

Index Fungorum: IF901619

Facesoffungi Number: FoF17276

Figs 1 , 12

###### Holotype.

MFLU 25-0020.

###### Etymology.

The compound epithet refers to the host genus, *Musa*, from which the species was isolated, and the location, Chiang Mai, where the fungus was collected.

###### Description.

Associated with leaf blight of *Musa* sp. ***Leaf blight*** elongated, pale brown to brown, surrounded with a dark brown margin. Sexual morph: Not observed. Asexual morph on PDA: ***Conidiomata*** 200–1000 µm diam. (x̄ = 450 µm, n = 10), semi-immersed, scattered or gregarious, globose to subglobose, dark brown to black, exuding creamy orange conidial mass. ***Setae*** not observed. ***Conidiophores*** formed directly from mycelium, hyaline, aseptate, smooth-walled. ***Conidiogenous cells*** 9–16 × 2–6 µm (x̄ = 13.2 × 4.2 µm, n = 10), hyaline, cylindrical to ampulliform or clavate, straight or flexuous, tapering towards the apex. ***Conidia*** 12–19 × 4.5–6 µm (x̄ = 15.7 × 5.4 µm, n = 30; L/W ratio = 2.9), hyaline, cylindrical to clavate, straight or slightly flexuous, smooth-walled, guttulate, aseptate, mostly with obtusely rounded ends (sometimes tapering towards one end). ***Appressoria*** not observed.

**Figure 12.**
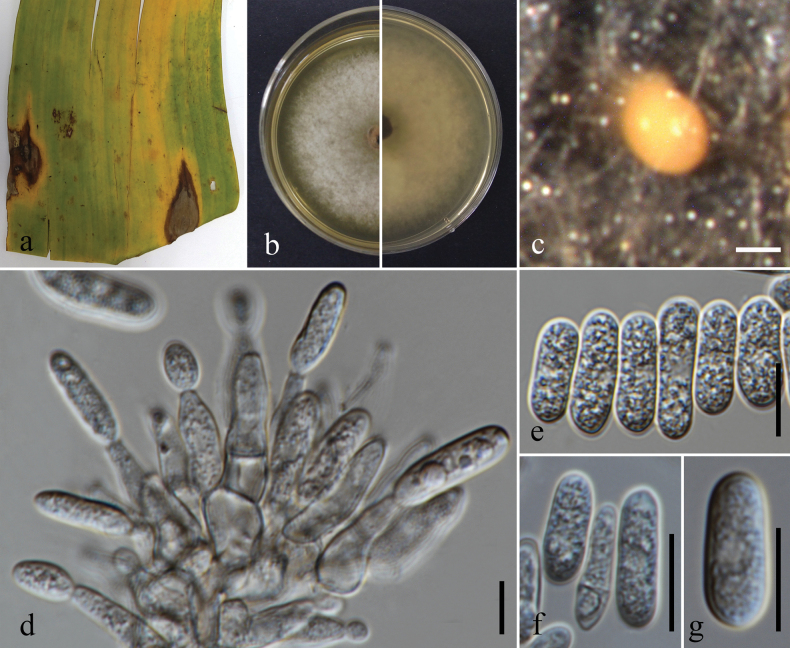
*Colletotrichummusichiangmaiense* (MFLU 25-0020, holotype). **a.** Leaf blight (*Musa* sp.); **b.** Top and reverse of colony on MEA after 5 d; **c.** Spore mass formation on MEA after 21 d; **d.** Conidiogenesis and developing conidia; **e–g.** Conidia. Scale bars: 500 μm (**c**); 10 μm (**d–g**).

###### Culture characteristics.

Colonies on MEA reaching approximately 75 mm diam. after 7 d of incubation at 25 °C; mycelium initially greyish white, becoming olivaceous brown with age, elevation flat, cottony, and fluffy, aerial and filamentous with an entire margin.

###### Specimen examined.

Thailand • Chiang Mai Province, Omkoi District, Yiang Piang Subdistrict, associated with leaf blight of *Musa* sp. (Musaceae), 16 Oct 2019, D. Gomdola DG385-T2 (MFLU 25-0020, ***holotype***), ex-type MFLUCC 25-0021.

###### GenBank accession numbers.

ITS = PV263308; *GAPDH* = PV290913; *ACT* = PV297888; and *TUB*2 = PV295628.

###### Known hosts, distributions, and lifestyles.

Associated with leaf blight of *Musa* sp. in Thailand (this study).

###### Notes.

Our isolate (MFLUCC 25-0021) matches the morphological species concept of *Colletotrichum* (Jayawardena et al. 2021a). It forms a distinct lineage (99% ML and 1.00 PP support; Fig. 1), separate from other *Colletotrichum* species, and does not group within any species complex. Based primarily on its phylogenetic placement and supported by morphological data, we designate our isolate as a new singleton species, *Colletotrichummusichiangmaiense*, associated with leaf blight of *Musa* sp. in Thailand.

## ﻿Discussion

The main aim of this study was to identify 20 isolates of *Colletotrichum* obtained from various diseased plants in Thailand based on morphological and phylogenetic species concepts. These isolates represent eight distinct species, encompassing two newly described taxa and six species that were reported from different hosts (accounting for 13 new host records). The *Colletotrichum* isolates obtained herein are distributed across three species complexes, except for one isolate that is a singleton species. The number of species and species complexes within this genus has been a topic of ongoing taxonomic debate (Marin-Felix et al. 2017; Bhunjun et al. 2021; Jayawardena et al. 2021a; Liu et al. 2022). Jayawardena et al. (2021a) recognized 14 species complexes in *Colletotrichum*. Liu et al. (2022) expanded this to 16 species complexes, while Talhinhas and Baroncelli (2023) proposed a further increase to 20 species complexes. Our updated phylogeny (Fig. 1), which was based on a large taxon sampling and the inclusion of seven informative gene regions (ITS and the genes encoding for *GAPDH*, *CHS*1, *ACT*, *TUB*2, *H*3, and *CAM*), identifies 15 species complexes within the genus. Including our newly introduced taxa (*C.dendrobii* and *C.musichiangmaiense*), there are currently 347 established species distributed in these 15 species complexes and 22 singleton species.

Based on our phylogenies, the species complexes are listed below in descending order of species richness, with the number of species in each complex indicated in brackets: *C.gloeosporioides* (84), *C.acutatum* (50), *C.boninense* (41), *C.graminicola-caudatum* (37), *C.destructivum* (25), *C.dematium* (19), *C.gigasporum* (15), *C.dracaenophilum* (14), *C.orchidearum* (13), *C.spaethianum* (12), *C.magnum* (10), *C.orbiculare* (9), *C.agaves* (7), *C.truncatum* (6), and *C.bambusicola* (5). Among these, the *C.gloeosporioides* species complex is the most speciose (Jayawardena et al. 2021a; Sui et al. 2024). Notably, in our study, 15 of the 20 isolates belong to five different species within this complex. Results obtained herein align with those of previous studies, highlighting the substantial diversity of the *C.gloeosporioides* species complex (Jayawardena et al. 2021a; Liu et al. 2022; Sui et al. 2024). Regarding singleton species, it is unclear in some cases whether they are truly singletons or belong to specific species complexes. In certain studies, some species are basal to specific species complexes and are thus classified as singletons, while in other studies, they group within a particular species complex (Sui et al. 2024). In this study, a few species that were initially introduced as singletons are now found to clade within specific species complexes. Accordingly, we classify the previously introduced singleton species, *C.hsienjenchang* and *C.metake*, within the *C.bambusicola* species complex, and *C.orchidophilum* within the *C.orbiculare* species complex.

*Colletotrichum* species exhibit life modes that allow them to manifest as saprobes, endophytes, and pathogens, depending on environmental conditions (Jayawardena et al. 2021a; Talhinhas and Baroncelli 2023). This ecological plasticity is significant, as certain species can switch from a symbiotic life mode (as endophytes or saprobes) to a pathogenic one under favorable conditions, such as when the host plant is stressed or weakened (Promputtha et al. 2007; Bhunjun et al. 2023). In this study, we isolated *Colletotrichum* species associated with foliar and pod diseases. Based on morphological characteristics and phylogenetic analyses of multiple gene regions, we established two novel species: *C.dendrobii* and *C.musichiangmaiense*. For *C.dendrobii*, the primary distinguishing feature compared to its sister taxon, *C.verruculosum* (IMI 45525), is its variable conidial shapes, a unique morphological trait not observed in *C.verruculosum*. The phylogenetic placement of *C.dendrobii* and the nucleotide differences between *C.dendrobii* and *C.verruculosum* further support its status as a new species. Regarding *C.musichiangmaiense*, phylogenetic analyses revealed that it does not group within any species complex and formed a completely distinct lineage, basal to the *C.gloeosporioides* species complex. Therefore, we confirmed *C.musichiangmaiense* as a new species distinct from any previously described taxa.

In addition to our two newly described species, we establish 13 new host records, representing six spp. isolated from various sites in Thailand, as listed below: *C.castaneae* (*Jasminumgrandiflorum*), *C.chrysophilum* (*Terminalia* sp.), *C.endophyticum* (leaf spots of *Artocarpusheterophyllus*, *Begonialuxurians*, *Castanopsis* sp.; and *Schefflera* sp.; leaf blight of *Ficusauriculata*; and leaf blotches of *Cassia* sp.), *C.fructicola* (leaf spots of *Castanea* sp., leaf blight of *Hedychium* sp., and leaf blotches of *Rhododendron* sp.), *C.jiangxiense*, and *C.schimae* (leaf spots of *Jasminum* sp.). These species have also been documented from other hosts in previous studies (Doyle et al. 2013; Ayvar-Serna et al. 2020; Fuentes-Aragón et al. 2020b; Liu et al. 2022; Pacheco-Esteva et al. 2022; Soares et al. 2022; Tang et al. 2023; Xu et al. 2023; Zhang et al. 2023a, 2023b, 2023c; Armand and Jayawardena 2024; Sui et al. 2024). Therefore, our findings further support the high diversity of *Colletotrichum* species, which are associated with a broad range of hosts.

*Colletotrichumcastaneae* was previously found associated with *Castaneamollissima* in China (Zhang et al. 2023a), while we found it associated with leaf spots of *Jasminumgrandiflorum* in Thailand. *Colletotrichumchrysophilum*, *C.endophyticum*, *C.fructicola*, and *C.jiangxiense* are reported to have a wide range of hosts, as mentioned in the taxonomy section. *Colletotrichumendophyticum* was initially identified as a leaf endophyte of *Pennisetumpurpureum* in Thailand (Manamgoda et al. 2013). Later, it was reported in both saprobic and pathogenic life modes, causing diseases such as anthracnose, fruit lesions, and leaf spots (Udayanga et al. 2013; Manawasinghe et al. 2022; Zhang et al. 2023c). In this study, *C.endophyticum* was isolated from six different hosts associated with foliar diseases: leaf spots of *Begonialuxurians* and *Schefflera* sp., leaf blight of *Ficusauriculata*, and leaf blotches of *Cassia* sp. Interestingly, it was also found associated with spots on dried dead leaves of *Castanopsis* sp. and *Artocarpusheterophyllus*. Since we isolated this fungus from spots of dry leaves, we speculate that it could colonize and infect living leaves, potentially leading to their senescence and death. However, since we did not conduct pathogenicity tests, we cannot confirm this hypothesis; the fungus could also be a saprobe on dead leaves. *Colletotrichumschimae* was initially reported only as an endophyte, with the suggestion that it might colonize host plants as a beneficial organism without causing disease (Liu et al. 2022; Liao et al. 2025). However, this species could be a potential opportunistic pathogen, as Sui et al. (2024) later reported it in a pathogenic mode from a few hosts. We report this species associated with leaf spots of *Jasminum* sp. in Thailand. Both *C.endophyticum* and *C.schimae* have a broader host range rather than being host-specific. Additionally, their life mode is not limited to endophytism, as initially presumed.

Interestingly, this study presents the first detailed description and illustration of the sexual morph of *Colletotrichumendophyticum*, offering new insights into its reproductive biology and lifecycle. So far, this species was solely reported in its asexual state as saprobes, endophytes, and pathogens. Therefore, this finding emphasizes the importance of examining both sexual and asexual morphs for a comprehensive understanding of *Colletotrichum* taxonomy, ecology, and reproductive strategies.

Over the past few years, extensive research has been conducted to identify and classify *Colletotrichum* taxa (Liu et al. 2022; Sui et al. 2024), and numerous novel species have been introduced (Manawasinghe et al. 2022; Armand et al. 2023; Zhang et al. 2023b; Aumentado et al. 2024; Sui et al. 2024; Zapata et al. 2024; Zhang et al. 2024). However, introducing new species within *Colletotrichum* requires careful consideration, as they often exhibit cryptic morphologies, minimal nucleotide differences, and frequent grouping within the same clade with minor genetic divergence. As a result, taxonomists may classify them as distinct species, although they might represent the same species. Recently, many species have been synonymized under older taxa (Aumentado et al. 2024; Sui et al. 2024).

In this study, we observed a similar pattern where species could potentially be synonymized. The type strains of both *Colletotrichumchrysophilum* [URM 7368; W.A.S. Vieira, W.G. Lima, M.P.S. Câmara, and V.P. Doyle] and *C.noveboracense* [CBS 146410; F. Khodadadi, P.L. Martin, V.P. Doyle, J.B. Gonzalez, and S.G. Aćimović] are positioned within the same clade, with strong statistical support (100% ML and 1.00 PP) (Fig. 2). All other strains of these two species also cluster together with 100% ML and 1.00 PP support. Despite this close phylogenetic relationship, we did not synonymize *C.noveboracense* with *C.chrysophilum* due to a difference of 1.4% (9/664 bp) in the mating type (Mat1-2) gene (*ApMat*) [*C.chrysophilum* URM 7368; KX094325 and *C.noveboracense* CBS 146410; MN640564]. Additionally, a slight nucleotide difference of 0.9% (2/219 bp) was observed in the *GAPDH* region. However, no differences were found in the ITS (495 bp), *ACT* (216 bp), *TUB2* (648 bp), and *CAL* (656 bp) regions between *C.chrysophilum* (URM 7368) and *C.noveboracense* (CBS 146410). Since we were unable to amplify the ApMat region from our strains, we could not directly compare our sequences with those of the type strains. Moreover, the observed difference in ApMat is relatively small and likely insufficient to justify segregation of the two species. Therefore, we have refrained from discussing their conspecificity (Vieira et al. 2017; Khodadadi et al. 2020).

To accurately identify and classify *Colletotrichum* species, a polyphasic approach, incorporating morphological traits with multigene phylogenetic analyses and geographical and/or ecological data, is recommended (Cai et al. 2009; Marin-Felix et al. 2017). Further studies focusing on the recollection and examination of problematic species are necessary to resolve their taxonomic placements and ensure a robust classification framework.

## Supplementary Material

XML Treatment for Colletotrichum schimae

XML Treatment for Colletotrichum castaneae

XML Treatment for Colletotrichum chrysophilum

XML Treatment for Colletotrichum endophyticum

XML Treatment for Colletotrichum fructicola

XML Treatment for Colletotrichum jiangxiense

XML Treatment for Colletotrichum dendrobii

XML Treatment for Colletotrichum musichiangmaiense

## ﻿Additional information

### Author ORCIDs

Deecksha Gomdola https://orcid.org/0000-0002-0817-1555

Rajesh Jeewon https://orcid.org/0000-0002-8563-957X

Eric H.C. McKenzie https://orcid.org/0000-0001-9400-7112

Ruvishika S. Jayawardena https://orcid.org/0000-0001-7702-4885

Fatimah Al-Otibi https://orcid.org/0000-0003-3629-5755

Xia Tang https://orcid.org/0000-0003-2705-604X

Kevin D. Hyde https://orcid.org/0000-0002-2191-0762

### Data availability

All data generated or analyzed during this study are included in this published article.

## Supplementary materials

Supplementary material 1 Isolates and their corresponding GenBank accession numbers Data type xlsx Explanation note Type, ex-type, and reference spe-cies are denoted with ^T^. Our isolates are in bold font. https://binary.pensoft.net/file/1366892This dataset is made available under the Open Database License (http://opendatacommons.org/licenses/odbl/1.0/). The Open Database License (ODbL) is a license agreement intended to allow users to freely share, modify, and use this Dataset while maintaining this same freedom for others, provided that the original source and author(s) are credited.Deecksha Gomdola, Rajesh Jeewon, Eric H.C. McKenzie, Ruvishika S. Jayawardena, Fatimah Al-Otibi, Xia Tang, Yong Wang, Kevin D. Hyde, Li Fu
